# Does regulation hold the key to optimizing lipopeptide production in *Pseudomonas* for biotechnology?

**DOI:** 10.3389/fbioe.2024.1363183

**Published:** 2024-02-27

**Authors:** Lu Zhou, Monica Höfte, Rosanna C. Hennessy

**Affiliations:** ^1^ Laboratory of Phytopathology, Department of Plants and Crops, Faculty of Bioscience Engineering, Ghent University, Ghent, Belgium; ^2^ Department of Plant and Environmental Sciences, University of Copenhagen, Copenhagen, Denmark

**Keywords:** *Pseudomonas*, specialized metabolites, lipopeptides, biosurfactants, antibiotics, regulation, bioengineering, bioprocessing

## Abstract

Lipopeptides (LPs) produced by *Pseudomonas* spp. are specialized metabolites with diverse structures and functions, including powerful biosurfactant and antimicrobial properties. Despite their enormous potential in environmental and industrial biotechnology, low yield and high production cost limit their practical use. While genome mining and functional genomics have identified a multitude of LP biosynthetic gene clusters, the regulatory mechanisms underlying their biosynthesis remain poorly understood. We propose that regulation holds the key to unlocking LP production in *Pseudomonas* for biotechnology. In this review, we summarize the structure and function of *Pseudomonas*-derived LPs and describe the molecular basis for their biosynthesis and regulation. We examine the global and specific regulator-driven mechanisms controlling LP synthesis including the influence of environmental signals. Understanding LP regulation is key to modulating production of these valuable compounds, both quantitatively and qualitatively, for industrial and environmental biotechnology.

## 1 Introduction

The climate and environmental challenges we face today are immense. Novel solutions encouraging the development of sustainable processes are urgently needed to support the green transition and contribute to the circular economy. One promising strategy to drive green technologies is the exploitation of microbes and their natural product diversity.

*Pseudomonas* species represent a large and diverse group of bacteria of significant importance for numerous biotechnological applications owing to unique characteristics of rapid growth, versatile utilization of sustainable carbon sources, high metabolic diversity and tolerance to extreme environments (Wang et al., 2020). Ubiquitous in nature, they perform key functions in complex ecosystems, e.g., plant surfaces, the rhizosphere, water, insects, humans and soils, including those with a history of chemical waste pollution (Nikel et al., 2014). *Pseudomonas* therefore possess an extensive application potential in some of the most challenging fields of industrial and environmental biotechnology, e.g., environmental restoration, plant growth promotion and protection, and the production of specialized metabolites (Nikel et al., 2014; Wang et al., 2020).

Bacterial specialized metabolites (SM), also called secondary metabolites or natural products, are high-value bioactive compounds with vast biotechnological potential. A significant challenge in the development of these compounds is the activation of SM pathways under laboratory conditions. Synthesis is catalyzed by mega-enzymatic complexes encoded by portions of bacterial genomes known as biosynthetic gene clusters (BGCs). Under standard laboratory conditions BGCs are often expressed at low levels or not at all, termed “silent” (Gram, 2015), so that bacterial genome sequences reveal a larger number of SM gene clusters than indicated by chemical analysis of culture extracts (Sanahuja et al., 2011; Reddy and Saravanan, 2013; Monfil and Casas-Flores, 2014). This complicates the lab-based isolation and characterization of SMs and hinders the development of bioprocesses for their production at scale.

*Pseudomonas* species are natural producers of a vast number of high-value bioactive compounds including biosurfactants such as rhamnolipids, and linear and cyclic lipopeptides (CLPs). Rhamnolipids and LPs are powerful biosurfactants and antibiotics with enormous potential for applications in medicine (e.g., antibiotics, antitumor, immunosuppressants, and cytotoxic agents acting on cancer cells), food and beverage (e.g., anti-spoilage agents, emulsifiers, foaming agents), cosmetics (e.g., antiaging and moisturizing products), textiles (e.g., preparation of fibers), cleaning products (e.g., household detergents and personal care products), bioremediation (e.g., degradation of xenobiotics, heavy metal removal from polluted soil) and agriculture (e.g., bioprotectants). They are attractive ecofriendly alternatives to chemical surfactants owing to their high specificity, biodegradability, low toxicity and effectiveness at extreme temperatures, pH and salinity (Abdel-Mawgoud et al., 2010). *Pseudomonas* biosurfactants have so far mainly been used in oil recovery and production, including as dispersants in the bioremediation of oil spills (Banat et al., 2000; De Almeida et al., 2016).

While LPs are produced by other bacteria, e. g., *Bacillus* species in addition to fungi, *Pseudomonas*-derived LPs in comparison represent a structurally and functionally large, and diverse group of compounds with broad-spectrum antimicrobial, antitumor, cytotoxic, immunosuppressant and surfactant properties (Raaijmakers et al., 2006; Geudens and Martins, 2018). LPs share a common structural blueprint consisting of a fatty acid tail coupled to the N-terminal of a short oligopeptide. In the case of CLPs, a lactone ring is formed between two amino acids resulting in a cyclic structure (Raaijmakers et al., 2006). The diverse structures and biological activities of linear lipopeptides (LLPs) and CLPs result from differences in fatty acid tail length and modifications in addition to the number, type, order and configuration of amino acids in the peptide moiety and lactone ring. LPs belong to the SM family of non-ribosomal peptides (NRPs) which unlike ribosomal peptides (RPs) are synthesized by enzymes capable of incorporating and subsequently modifying both proteinogenic and so called unusual non-proteinogenic amino acids into the oligopeptide. As a result, LPs display an increased level of diversity and multifunctionality.

High cost of production and low yields are major bottlenecks restricting the development and application of LPs. We propose that unravelling the regulatory networks of global and specific regulators underpinning LP synthesis in *Pseudomonas* holds the key to unlocking production for biotechnology. While numerous regulatory genes have been identified in several LP-producing strains, knowledge of the regulatory mechanisms and critically, the environmental signals controlling production remains in its infancy.

**FIGURE 1:**
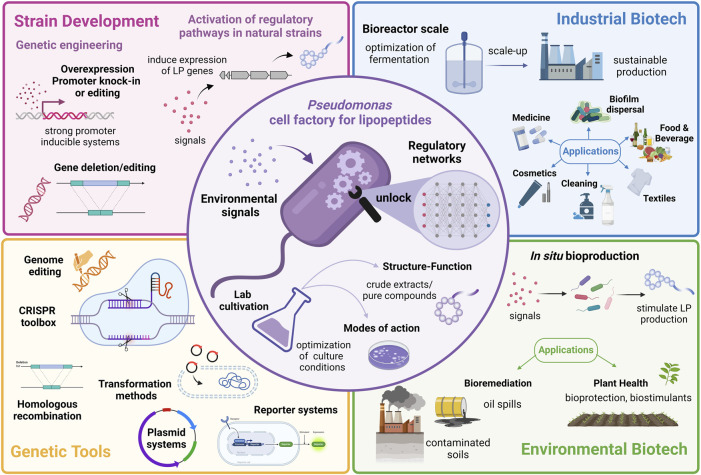
Graphical abstract showing strategies to exploit the regulatory pathways controlling lipopeptide production in *Pseudomonas* for applications in industrial and environmental biotechnology. Created with BioRender.com.

In this review, we summarize the structure and function of *Pseudomonas*-LPs and examine the regulatory mechanisms and environmental signals, i.e., bi*otic and abiotic factors* influencing their synthesis. Finally, the challenges and opportunities of exploiting regulation to optimize LP production in *Pseudomonas* will be discussed. A graphical summary of the review is presented in Figure 1.

## 2 Structural diversity and phylogeny

The two main lineages of the genus *Pseudomonas* (*Pseudomonas aeruginosa* and *Pseudomonas fluorescens* lineages) both naturally produce biosurfactants, but strains belonging to the *P. aeruginosa* lineage produce rhamnolipids whereas members of the *P. fluorescens* lineage produce LPs. Based on their structures *Pseudomonas* LPs can be divided into at least 14 different families that differ in oligopeptide length (L) ranging from 8 to 25 amino-acids and macrocycle length (M) ranging from 0 in LLPs to 4 to 9 amino acids in CLPs (Table 1).

**TABLE 1:** Structural diversity of *Pseudomonas* lipopeptides and producing taxonomic groups and subgroups.

Structure			TAXONOMY^b^		
Family	L:M tag^a^	Type	Group	Subgroup	Lipopeptides^c^
Factin	8:0	lineair	*syringae*	phylogroup 1, 2, 5, 6, 10	syringafactin
				phylogroup 7, 8, 9, 11	cichofactin
			*fluorescens*	*asplenii*	virginiafactin
			*fluorescens*	*corrugata*	thanafactin
			*putida*	*vranovensis, cremoricolorata*	cichofactin, syringafactin
Bananamide	8:6	cyclic	*fluorescens*	*koreensis*	bananamide A-C, bananamide D-G, MDN-0066, prosekin, pseudofactin
Viscosin	9:7	cyclic	*fluorescens*	*fluorescens, chlororaphis, gessardii*	viscosin, WLIP, viscosinamide, pseudodesmin, pseudophomin, massetolide
			*putida*	*wayambapalatensis, xanthosomae*	WLIP
Mycin	9:9	cyclic	*fluorescens*	*corrugata, mandelii, asplenii*	cormycin, syringomycin, thanamycin, syringotoxin, nunamycin, keanumycin
			*syringae*	phylogroup 2, 8, 10, 11	syringomycin, syringotoxin, syringostatin, pseudomycin
Poaeamide	10:8	cyclic	*fluorescens*	*fluorescens*	Poaeamide, PPZPM
Orfamide	10:8	cyclic	*fluorescens*	*protegens*	orfamide A-G
Cocoyamide	11:5	cyclic	*fluorescens*	*koreensis*	cocoyamide/gacamide
Amphisin	11:9	cyclic	*fluorescens*	*koreensis*	arthrofactin, lokisin, anikasin, amphisin, hodersin, milkisin, tensin, nepenthensin, oakridgin
Putisolvin	12:4	cyclic	*putida*	*reidholzensis, capeferrum, vlassakiae*	putisolvin I-III
Asplenin	13:8	cyclic	*fluorescens*	*asplenii*	asplenin
Entolysin	14:5	cyclic	*putida*	*mosselii*	entolysin
Xantholysin	14:8	cyclic	*putida*	*mosselii*	xantholysin
Tolaasin	18:5	cyclic	*fluorescens*	*fluorescens, protegens*	tolaasin I, tolaasin F, sessilin A
Peptin	19:5	cyclic	*fluorescens*	*asplenii*	fuscopeptin, jessinipeptin
	22:5	cyclic	*fluorescens*	*corrugata, mandelii*	corpeptin, nunapeptin, thanapeptin, braspeptin
	22:0	lineair	*fluorescens*	*corrugata*	sclerosin
	22:5	cyclic	*syringae*	phylogroup 2	syringopeptin SP22
	22:8	cyclic	*syringae*	phylogroup 8, 11	cichopeptin, cichorinotoxin
	25:8	cyclic	*syringae*	phylogroup 2, 10	syringopeptin SP25

^a^ L: number of AA, in the oligopeptide, M: number of AA, in the macrocycle.

^b^ *See* (Girard et al., 2021) *for a recent update on Pseudomonas taxonomy*.

^c^ *See* (Girard et al., 2020; Götze and Stallforth, 2020; Cesa-Luna et al., 2023) *and* *https://rhizoclip.be/* *for chemical structures*.

Within the *P. fluorescens* lineage, LP producers reside in the *P. fluorescens, Pseudomonas putida* and *Pseudomonas syringae* groups (Cesa-Luna et al., 2023). The *P. fluorescens* group is further divided into nine major subgroups (*P*. *mandelii, P. jessenii, Pseudomonas koreensis, Pseudomonas corrugata, P. fluorescens, P. gessardii, Pseudomonas chlororaphis*, and *Pseudomonas protegens*) (Girard et al., 2021) comprising both beneficial bacteria and plant pathogens. The *P. syringae* group, harboring many plant pathogens, is further divided into 13 phylogroups (Berge et al., 2014). LP producers are found in phylogroups 2, 8, 10 and 11 and they typically produce two types of CLPs, respectively from the Mycin and Peptin families, in addition to a LLP from the Factin family. Single Factin producers are found in various other *P. syringae* phylogroups (Bricout et al., 2022) (Table 1). Strains producing Mycin and Peptin variants are also found in the *P. fluorescens* group, notably in the *P. asplenii*, *P. mandelii* and *P. corrugata* subgroups (Girard et al., 2020). Most other strains belonging to the *P. putida* and *P. fluorescens* group are LP mono-producers, and they produce one or more LP variants that belong to a single LP family. There is a strong correlation between LP type produced and species diversification (Cesa-Luna et al., 2023) with a few notable exceptions, strains that have obtained an LP biosynthetic gene cluster by horizontal transfer. To date, roughly 120 LPs have been described in strains inhabiting diverse environments, but LP producers are often associated with plants.

## 3 Function and applications

*Pseudomonas* LPs display an incredible architectural and chemical diversity and consequently exhibit a range of different biological activities. Here, we briefly present the various biological properties and functions of LPs and discuss their potential biotechnological applications. More detailed information on the natural functions and roles of *Pseudomonas* LPs is provided in various reviews (D'Aes et al., 2010; Raaijmakers et al., 2010; Geudens and Martins, 2018; Girard et al., 2020; Götze and Stallforth, 2020).

### 3.1 LPs as biosurfactants

LPs are surface-active molecules also called biosurfactants. Biosurfactants help to condition the producing strain’s environment by supporting various processes including bacterial motility, attachment and colonization of surfaces, biofilm development and access to nutrients and water (Nybroe and Sorensen, 2004; D'Aes et al., 2010; Raaijmakers et al., 2010; Geudens and Martins, 2018; Bricout et al., 2022).

Various lab-based studies show a strong correlation between LP production and swarming motility when comparing wild-type and LP-deficient mutant strains (de Bruijn et al., 2008; de Bruijn and Raaijmakers, 2009; Geudens and Martins, 2018). The ability of LPs to alter surface tension and viscosity is determined by their structural properties (D'Aes et al., 2010). In the amphisin producer *Pseudomonas* sp. DSS73, *amsY* and *gacS* mutant strains are unable to swarm on soft agar and as expected swarming motility is restored by the addition of amphisin but also viscosin, tensin and serrawettin from *Serratia liquefaciens* (Andersen, 2003). In contrast, synthetic surfactants were unable to complement the non-motile phenotype indicating that unique physiochemical properties relating to the chemical structure of LPs contribute to bacterial movement (Andersen, 2003). The diverse structures and physiochemical properties of LPs make them attractive alternatives to chemical surfactants for numerous industrial applications (Ceresa et al., 2023).

One of the most effective biosurfactants reported to date is surfactin from *Bacillus subtilis c*apable of lowering the surface tension of water from 71 to 27 mN/n at a critical micelle concentration (CMC) of 20 µM (Yeh et al., 2005). Viscosin also shows strong surface activity reducing water surface tension to 25 mN/n at CMC of 4 μg/mL (Nybroe and Sorensen, 2004). Similarly, viscosinamide reduces water surface tension to 27 mN/n however no CMC values are available (Nybroe and Sorensen, 2004). Syringomycin and syringopeptin reduce water surface tension in ranges of 31–35 mN/n comparable to values of 31.5 mN/n and 38 mN/n recorded for putisolvin II and tolaasin, respectively (Hutchison and Johnstone, 1993; Nybroe and Sorensen, 2004; Janek et al., 2010). For most *Pseudomonas* LPs however information on physiochemical properties, e.g., *surface reduction activities and CMC values, foaming capacity, emulsifying activity and compound stability* are lacking.

*In situ* studies support the role of LPs in motility and colonization of specific habitats, e.g., plant material or fungal tissues. Massetolide A contributes to colonization of tomato roots by *P. lactis* (*fluorescens*) SS101, viscosin is required for colonizing broccoli florets by a pectolytic strain of *P. fluorescens*, cichofactin is essential for *Pseudomonas cichorii* to colonize lettuce leaves, while *Pseudomonas* sp. DSS73 uses amphisin to colonize sugar beet seeds and roots (Hildebrand et al., 1998; Nielsen and Sorensen, 2003; Tran et al., 2007; Pauwelyn et al., 2013). Other LPs, for example, poaeamide from *Pseudomonas poae* RE*1-1-13 and putisolvin from *P. putida* 257 do not positively contribute to rhizosphere competence as no differences in plant root colonization are observed between WT and biosurfactant deficient mutant strains (Kruijt et al., 2009; Zachow et al., 2015).

For many plant-associated LP-producers, biosurfactant-assisted motility is a key determinant for successful rhizosphere and phyllosphere colonization, where in addition to plant material, LPs also mediate interactions with fungi. In the phytopathogen *Ralstonia solanacearum*, the LP ralsolamycin (RM) facilitates fungal tissue invasion by inducing the formation of fungal survival structures known as chlamydospores in *Aspergillus flavus* (Spraker et al., 2016). An RM deficient *rmyA* mutant cannot induce chlamydospore formation and shows reduced hyphal invasion (Spraker et al., 2016). Subsequently (Venkatesh et al., 2022), demonstrated that compared to the *rmyA* mutant, WT *R. solanacearum* is internalized in chlamydospores during co-culture with *A. flavus* and shows increased fitness under starvation and cold stress. Phylogenetically distinct bacteria (not associated with endofungal lifestyles) including the nitrogen-fixing bacterium *Herbaspirillium seropedicae* were also shown to colonize chlamydospores of *A. flavus* when treated with WT supernatants further confirming the role of RM in facilitating fungal invasion (Venkatesh et al., 2022). Such knowledge could be translated to stimulate diverse endofungal interactions for improved ecosystem services, e.g., nitrogen fixation (Venkatesh et al., 2022). While a number of other LPs including the *Pseudomonas* CLPs viscosinamide and tensin can induce fungal survival structures, their role in fungal invasion and endosymbiosis is currently unknown (Nielsen et al., 1999; Nielsen et al., 2000; Venkatesh et al., 2022). More information on interactions with fungi is described in Section 3.2.2.

Biosurfactants also regulate biofilm development with a number of CLPs shown to promote biofilm formation (massetolide A, sessilin and xantholysin) and others involved in biofilm dispersal (arthrofactin, orfamide and putisolvin) (Kuiper et al., 2004; Bonnichsen et al., 2015). In *P. sessilinigenes* CMR12a, orfamides are indispensable for swarming motility, while sessilin is important for biofilm formation (D’aes et al., 2014). Based on contrasting studies, viscosin is proposed to mediate both biofilm formation and dispersal (de Bruijn et al., 2007; Bonnichsen et al., 2015). Viscosin-mediated biofilm dispersal is dependent on carbon starvation and by microscopic analysis it was observed that cells exhibiting high *viscA* (required for viscosin biosynthesis) expression levels were leaving biofilms, further supporting the role of LPs in motility. However, information on the mechanisms of LP-mediated biofilm development and dispersal, particularly *in situ* is limited and could benefit from more temporal studies examining LP function during biofilm lifecycles. For the producing bacteria, the ability to disperse is an important escape function under unfavorable nutrient conditions to support their spread throughout the environment and enable the colonization of new niches. From an industrial perspective, LPs displaying roles in biofilm formation could be used for bulk chemical production using biofilm fermentations whereas dispersal functions could be exploited as surface-coating agents or used in disinfectant formulations (Bonnichsen et al., 2015; Leonov et al., 2021).

Additional biological properties of LPs include the chelation of metal ions and xenobiotic degradation, e.g., petroleum hydrocarbons and pesticides (Nybroe and Sorensen, 2004; Raaijmakers et al., 2010; de Cássia et al., 2014; Raj et al., 2021). Research on biosurfactants as bioremediation agents to clean up contaminated soils is largely limited to rhamnolipids or *Pseudomonas*-leachates containing uncharacterized LPs (Sekhon Randhawa and Rahman, 2014; Sun et al., 2021). The CLPs viscosin, amphisin, massetolide A and putisolvin can emulsify alkane hydrocarbons such as n-hexadecane (Bak et al., 2015). Viscosin was also shown to stimulate alkane mineralization by a diesel-degrading bacterial consortium however the activity of the CLP was short-lived due to rapid (likely microbial) degradation (Bak et al., 2015). While LPs are proposed to chelate heavy metals and degrade insoluble hydrocarbons to increase their bioavailability and/or detoxify polluted soils for protection against toxicants, roles of LPs in such processes remain unclarified (Raaijmakers et al., 2010; Gutiérrez-Chávez et al., 2021).

### 3.2 LPs as antimicrobial compounds

*Pseudomonas*-CLPs display broad-spectrum antimicrobial properties exerting effects against bacteria, fungi, oomycetes and viruses as previously reviewed by (Nielsen et al., 2002; Geudens and Martins, 2018; Girard et al., 2020; Oni et al., 2022).

#### 3.2.1 Antibacterial activity

*Pseudomonas*-LPs show antagonistic activities against diverse Gram-positive and Gram-negative bacteria including human, plant and animal pathogens (Raaijmakers et al., 2006; Geudens and Martins, 2018). In general, Gram-positive bacteria are more susceptible, for example; viscosin, massetolide A and syringomycins 22A and E inhibit *Mycobacteria spp,*; amphisin, syringopeptins 22A and 25A in addition to corpeptin and WLIP are active against *Bacillus* spp.; syringopeptins also display inhibitory effects active against *Rhodococcus* spp., and *Micrococcus* spp. (Nybroe and Sorensen, 2004; Geudens and Martins, 2018), while viscosin and tensin are active against *Streptomyces scabies* (Pacheco-Moreno et al., 2021). Medipeptin A, produced by *Pseudomonas mediterranea* EDOX is active against *Staphylococcus aureus* and *Bacillus cereus* with a MIC of 8 μg/mL and against *Micrococcus flavus* with a MIC of 2 μg/mL (Zhou et al., 2021). Medipeptin A exerts its activity against *S. aureus* by binding to the cell wall polymer lipoteichoic acid and the cell wall precursor lipid II and by forming pores in membranes (Zhou et al., 2021). Jessenipeptin and mupirocin (a polyketide antibiotic), co-produced by *Pseudomonas* sp. QS1027, show synergistic activity against methicillin-resistant *S. aureus* (Arp et al., 2018).

Fewer LPs show activity against Gram-negative bacteria possibly due to the inability of LPs to access the outer membrane or peptidoglycan layer of Gram-negative cell walls (Nybroe and Sorensen, 2004). For example, syringomycins E and syringopeptin 25A show inhibitory effects against *P. syringae* but only upon treatment with lysozyme (Fogliano et al., 2002). Tolaasin I and WLIP show low inhibition of Gram-negatives whereas LPs of the xantholysin group are active against various Gram-negative and Gram-positive bacteria (Li et al., 2013). Interestingly, Rainey et al. (1991) reported tolaasin resistant Gram-negative bacteria (*Pseudomonas reactans*, *P. putida* and *E. coli*) become sensitive to the toxin when challenged by tolaasin and polymyxin B, highlighting the importance of synergistic activities of compounds during antagonism.

#### 3.2.2 Antifungal and anti-oomycete activities

Extensive research has focused on the inhibitory activities of *Pseudomonas*-LPs against numerous fungi, oomycetes and yeasts (Nybroe and Sorensen, 2004; Raaijmakers et al., 2006; Geudens and Martins, 2018; Omoboye et al., 2019b; Oni et al., 2022). One of the most active compounds is tolaasin, and 18:5 CLP produced by the mushroom pathogens *Pseudomonas tolaasii* and *P. costantinii* (Scherlach et al., 2013). These bacteria cause brown blotch disease characterized by dark brown lesions on the fruiting bodies of various mushroom species including the button mushroom *Agaricus bisporus*, the oyster mushroom *Pleurotus ostreatus* and shiitake (*Lentinula edodes*) (Soler-Rivas et al., 1999; Osdaghi et al., 2019). Tolaasin I is the main virulence factor of *P. tolaasii* and toxic towards mushrooms (Rainey et al., 1991; Lo Cantore et al., 2006; Andolfi et al., 2008). Tolaasin disrupts the fungal membrane by forming trans-membrane pores, allowing the producing bacteria access to cell nutrients. Tolaasin I is most active and also shows strong antimicrobial activity against other Basidiomycetes (Bassarello et al., 2004; Lo Cantore et al., 2006), a variety of Ascomycetes (Lo Cantore et al., 2006; Andolfi et al., 2008; Ferrarini et al., 2022a), and Oomycetes (Lo Cantore et al., 2006; Andolfi et al., 2008; Ferrarini et al., 2022a), with minimum inhibitory quantities (MIQs) ranging from 0.08 µg for *R. solani* and *A. bisporus* to 0.64 µg for some plant pathogenic Ascomycetes. Yeast-like fungi that cause diseases in animals and humans were less sensitive to tolaasin I (Andolfi et al., 2008). Ferrarini et al. (2022a) recently showed that within the Oomycetes *P. nicotianae* (EC_50_ = 5.6 µM) is considerably less sensitive to tolaasin I than *Pythium myriotylum* (EC_50_ = 0.30 µM in the absence of sterols).

A variant of tolaasin, called sessilin, is made by the well-studied biocontrol strain *P. sessilinigenes* CMR12a. This strain, isolated from cocoyam roots in Cameroon, also produces the 10:8 CLP orfamide (D’aes et al., 2014). Sessilin and orfamide contribute to the control of the cocoyam root rot disease caused by the Oomycete pathogen *P. myriotylum*, with sessilin showing the strongest inhibitory activity (Oni et al., 2019b).

Members of the Viscosin, Orfamide, Poaeamide and Putisolvin family (see Cesa-Luna et al. (2023) and Supplementary Table S1 for producing strains) cause immobilization and lysis of zoospores produced by oomycetes at concentrations around 25 µM (Gross et al., 2007; Raaijmakers et al., 2010; Zachow et al., 2015; Ma et al., 2016a). Microscopy studies show that various LPs of the Viscosin, Bananamide and Amphisin family induce morphological changes in fungi and oomycetes; viscosinamide increases branching, hyphal swelling and rosette formation in *R. solani* in addition to reduced mitochondria activities and changes in mitochondria morphology (Nielsen et al., 1999; Raaijmakers et al., 2006). Comparable microscopic observations have been made for viscosinamide against *Pythium ultimum* (Nielsen et al., 1999), while *P. myriotylum* challenged by pseudodesmin, viscosinamide and WLIP at concentrations ranging from 100 nm to 50 µM shows hyphal disintegration with pseudodesmin showing reduced hyphal branching at 1 and 50 µM while viscosinamide distorts fungal hyphae causing lysis at concentrations below 50 µM (Oni et al., 2020a). Increased branching and swelling also occurs for fungal hyphae treated with tensin (Nielsen et al., 2000). Bananamides target *P. oryzae* causing extensive hyphal branching, leakage and vacuolation (Omoboye et al., 2019a). Entolysin A and B permeabilize the membranes of *Pyricularia oryzae* and *B. cinerea* spores and mycelium as revealed by propidium iodide assays, starting at concentrations of 32 μM, with entolysin B being more active than entolysin A (Muangkaew et al., 2023). Antifungal activity of xantholysin has been tested using mutants, suggesting some activity against Ascomycetes, but this remains unconfirmed with pure compounds (Li et al., 2013).

Mycin and Peptin variants produced by specific strains from the *P. syringae* and the *P. fluorescens* group show interesting antifungal and anti-oomycete activity (Girard et al., 2020). Activity has mainly been shown by using mutants impaired in LP production. Only a few studies have used pure compounds, reflecting the difficulties in obtaining enough pure compound for biological assays. Keanumycin A, from *Pseudomonas* sp. QS1027, shows strong antifungal activity against human fungal pathogens including *Candida* spp. (MIC = 0.86 µM) and was extremely effective against *B. cinerea* (0.07 µM, 80 μg/L) (Götze et al., 2023). Also syringomycin E, syringotoxin B and syringostatin A, produced by strains of *P. syringae* pv. *syringae*, show fungicidal activity against *Candida* spp. (Sorensen et al., 1996). Nunamycin and nunapeptin are produced by *P. nunensis* In5, isolated from a potato soil suppressive against *R. solani* AG3 in southern Greenland (Michelsen and Stougaard, 2011; Ntana et al., 2023). Nunamycin production is required to inhibit *R. solani* growth in co-culture on agar plates and in a soil microcosm where disease incidence in tomato seedlings was significantly increased in a nunamycin mutant strain compared to the WT (Michelsen et al., 2015b). By using purified CLPs it was shown that nunamycin is more active against *R. solani* compared to *Pythium aphanidermatum* which appears more sensitive to nunapeptin. Thanamycin and thanapeptin are produced by *Pseudomonas* sp. SH-C52, a well-studied biocontrol agent isolated from sugar beet plants grown in a soil naturally suppressive to *R. solani* (Van Der Voort et al., 2015)**.** Thanamycin has antimicrobial activity against *R. solani* and a range of other fungi, while some derivatives of thanapeptin have anti-oomycete activity. Sclerosin, a 22:0 LLP from the Peptin family made by *Pseudomonas brassicacearum* DF41 isolated from canola roots, has activity against the fungal pathogen *Sclerotinia sclerotiorum* (Berry et al., 2012). Mutant analysis has revealed activity against *Botryosphaeria dothidea* for braspeptin, made by *Pseudomonas* sp. 11K1 (Zhao et al., 2019). Other Peptin family members such as syringopeptins show strong activity against various yeasts (Grgurina et al., 1996; Lavermicocca et al., 1997), while Fuscopeptin A and syringopeptin 22-A are toxic to *B. cinerea* at 20 µM.

Synergistic activities of LP product mixtures and/or with other molecules appear key to fungal antagonism. For example, *P. syringae* pv*. syringae* strain B359 secretes syringomycin E and syringomycin 25A in tandem with cell-wall degrading enzymes to inhibit fungal growth (Fogliano et al., 2002). The toxins show inhibitory activity against numerous fungi whereby antifungal activity is enhanced by the addition of purified enzymes and *in vivo* during co-culture with *Trichoderma atroviride*. Interestingly, syringomycin 25A is more potent against fungi in the presence of hydrolytic enzymes whereas syringomycin E shows greater inhibition of fungal growth and spore germination without hydrolytic enzymes (Fogliano et al., 2002). Other examples of antifungal synergism include orfamide and sessilin production in *P. sessilinigenes* CMR12a as well as nunamycin and nunapeptin production in *P. nunensis* In5 where co-production of the compounds increases inhibition of fungal growth. Crude extracts of *P. nunensis* In5 show greater inhibition against *R. solani* and *P. aphanidermatum* than pure compounds indicating the importance of synergistic activities of CLPs during interactions with pathogens and likely other organisms (Michelsen et al., 2015a). Moreover, sessilin and orfamide act additively in the biological control of the basidiomycete pathogen *R. solani* in bean and cabbage (Olorunleke et al., 2015). Interestingly, orfamide A and sessilin show no antifungal activity against *R. solani* when applied individually whereas nunamycin and nunapeptin target *R. solani* and *P. aphanidermatum* respectively (Michelsen et al., 2015a; Olorunleke et al., 2015).

More recently, viscosin-like CLPs produced by *P. cichorii* (identification based on 16S rRNA gene and probably not correct) demonstrated antagonistic activity against the human and vertebrate pathogens *Aspergillus fumigatus* and *Batrachochytrium dendrobatidis* (Martin et al., 2019). Using chemical imaging viscosin and massetolide were detected at the fungal inhibition zone suggesting synergistic activities may enhance their antifungal properties. Lab-based assays showing inhibition effects against both pathogens were however only reported for purified viscosin (Martin et al., 2019). In general, the mechanisms underlying synergistic interactions of LPs remain largely unknown.

#### 3.2.3 Antiviral activity

Antiviral activity is documented for viscosin against bronchitis virus and human-pathogenic viruses but the mechanism of viral inactivation is unknown (Raaijmakers et al., 2006). An analogue of xantholysin (MA026) from *Pseudomonas* sp. RtlB025 suppresses infectious hematopoietic necrosis virus (IHNV) and displays antiviral activity against hepatitis C virus (HCV) infection (Shimura et al., 2013). Following the global corona pandemic there has been considerable interest in developing diverse antiviral drugs. Xia et al. (2021) modelled the ability of diverse LPs to target coronavirus replication and transcription machinery and found *Pseudomonas*-derived ferrocin A and iron-chelating ferrocin A to be the best performing molecules (Xia et al., 2021). However, no studies examining the biological activities of *Pseudomonas* LPs against COVID-19 and SARS-CoV-2 exist.

### 3.3 LPs as cytotoxic agents

LPs also possess anti-proliferative activities against different cancer cell lines including viscosin (breast and prostate cancer cell lines) (Siani et al., 2008), xantholysin A (Pascual et al., 2014) and MDN-0066 (Cautain et al., 2015) (kidney tumor cell lines), pseudofactin II (melanoma cell lines) (Janek et al., 2013) and nunamycin/nunapeptin (mantle cell lymphoma, melanoma cell lines, T-cells leukemia) (Michelsen et al., 2015a). Accurate comparison of LP cytotoxic activities is challenged by the lack of standardization across assays. Similar to antimicrobial testing crude extracts over purified compounds are often used making the interpretation of results difficult as the bioactivities observed may derive from other compounds or synergistic activities between compounds. This has been observed in *P. nunensis* In5, where increased cytotoxic activity was seen when purified nunamycin and nunapeptin are mixed instead of applied individually (Michelsen et al., 2015a).

### 3.4 Interactions with plants

*Pseudomonas* LP producers are implicated in both positive and negative interactions with plants. Phytopathogenic strains typically co-produce phytotoxic CLPs from the Mycin and Peptin family, which act as virulence factors and form pores in plant membranes causing electrolyte leakage and necrosis. They usually also co-produce a third LLP or CLP not directly involved in virulence but instead aiding in plant tissue colonization. Phytopathogenic *Pseudomonas* LP producing strains taxonomically belong to the *P. syringae* group, or to the *P. asplenii* and *P. corrugata* subgroup of the *P. fluorescens* group (Girard et al., 2020). Phytotoxic CLPs from the Mycin and Peptin family also have strong antimicrobial activity (see above), demonstrating a dual role in pathogenicity and antagonism against competitors. Strains belonging to the *P. corrugata* subgroup can behave as plant pathogens, causing pit necrosis on tomato and pepper, but also show strong biological control activity against plant pathogens. They are often isolated from the roots and rhizosphere of non-diseased plants and from bulk soil (Catara, 2007; Gislason and de Kievit, 2020). Cormycin and corpeptin produced by *P. corrugata* double up as phytotoxic compounds and antimicrobial molecules against bacterial and fungal pathogens. *Pseudomonas* sp. SH-C52 (Mendes et al., 2011) (see above) also belongs to the *P. corrugata* subgroup and produces Mycin and Peptin-type CLPs with antifungal and anti-oomycete activity, indicating that there is no clear-cut line between plant pathogens and beneficials in these groups (Girard et al., 2020).

Disease suppressive soils are a rich source of LP-producing *Pseudomonas* strains. The potent biocontrol agents *P. nunensis* In5 (Michelsen and Stougaard, 2011) and *Pseudomonas* sp. SH-52 have been obtained from *R. solani* suppressive soils. Irrigation is known to protect potato tubers against the scab pathogen *S. scabies.* Microbiome analysis revealed that irrigated potato field had a larger proportion of Pseudomonadales bacteria than a non-irrigated potato field and that the presence of biosynthetic gene clusters encoding CLPs was positively correlated with disease suppression. Tensin, an 11:9 amphisin family CLP proved to be key determinant of *in planta* inhibition of potato scab in glasshouse trials (Pacheco-Moreno et al., 2021). Likewise, *Pseudomonas* strains able to produce CLPs belonging to 11 different families are dominant in the rhizosphere of cocoyam plants grown in a tropical soil suppressive to the cocoyam root rot disease caused by *P. myriotylum* (Oni et al., 2019a; Oni et al., 2020b).

*Pseudomonas* LP-producers demonstrating fungal and/or oomycete antagonism have gained considerable interest as candidates for controlling plant diseases. A detailed overview of *Pseudomonas* LP-mediated biocontrol is given by (D'Aes et al., 2010; Raaijmakers et al., 2010; Höfte, 2021; Oni et al., 2022). However, it is important to note that many studies are centered on the collection of data from lab-based experiments with only a handful of reports linking lab data to microcosm- or field-based studies. For example, for the viscosinamide producer *P. fluorescens* DR54 (Nielsen and Sorensen, 2003) a strong correlation between the ability of DR54 to inhibit growth of *R*. *solani* and *P*. *ultimum* in co-culture and during colonization of the rhizosphere of germinating sugar beet using plant-soil microcosms was observed. The study highlights the multifunctionality of CLPs with antibiotic and surfactant properties enabling the producing strain to condition its environment for successful rhizosphere colonization.

As wetting agents LPs can increase the solubility of nutrients and hydrophobic substrates for the producing strain (Nybroe and Sorensen, 2004; D'Aes et al., 2010; Mavrodi et al., 2010). For example, Bunster et al. (1989) reported that compared to a non-surface active *Pseudomonas* strain, surface-active isolates of *P. fluorescens* and *P. putida* increased the wetness of wheat leaves. Likewise, syringafactins are strong biosurfactants exerting hygroscopic activities to attract water vapor from the atmosphere increasing water availability and reducing water stress in *P. syringae* pv. syringae B728a on dry leaves and the apoplast of bean (Burch et al., 2014). Increasing the availability of nutrients and water potentially offers LP-producers a competitive advantage against other microbes including phytopathogens and thus may indirectly contribute to plant disease management by reducing pathogen growth delaying disease onset in the host plant (Nybroe and Sorensen, 2004).

A number of CLPs including massetolide A (Tran et al., 2007), sessilin, orfamide (Ma et al., 2016b; Ma et al., 2017), WLIP, lokisin and entolysin (Omoboye et al., 2019b) are involved in the induction of systemic resistance in plants. This type of resistance is systemically expressed rendering plants less susceptible to subsequent infection with pathogens (Pršić and Ongena, 2020). These studies are typically conducted with CLP mutants and CLP crude extracts with only a few studies demonstrating induction of ISR by pure compounds. Application of purified massetolide A at a concentration of 44 µM to tomato leaves or roots reduced the lesion area caused by *Phytopthora infestans*, but did not reduce disease incidence (Tran et al., 2007). Pure orfamide triggered ISR against *R. solani* web blight in bean at concentrations ranging from 0.001 to 0.1 µM (Ma et al., 2016b), while 25 µM orfamide was needed to elicit resistance to *Cochliobolus miyabeanus* in rice (Ma et al., 2017) suggesting different mechanisms involved.

### 3.5 Interactions with other eukaryotes

While protists stimulate beneficial plant-microbe interactions and contribute important functions, *e.g., nutrient cycling and pathogen removal* they are major bacterial predators (Gao et al., 2019; Bahroun et al., 2021; Guo et al., 2021; Hawxhurst et al., 2023). One predation defense mechanism used by bacteria involves LPs. Using a combination of wild-type and mutant *P. fluorescens* strains, Mazzola et al. (2009) showed that massetolide and viscosin protect bacteria against predation by the amoeba *Naegleria americana*, with the predator showing a greater sensitivity to viscosin. LP-producers showed better persistence and protection in soil against the predator but the effect was only temporal (Mazzola et al., 2009). *P. nunensis* 4Aze was co-isolated with the social amoeba *Polyspondyllium pallidum* RM1 from forest soil. This strain produces keanumycin D, and nunapeptin B and C with suppressive activities against amoebal predators and the bacterivorous nematode *Oscheius myriophilus*, highlighting the broad-spectrum activity of LPs and underexplored anti-predator function of these compounds (Pflanze et al., 2023) Also the keanumycins produced by *Pseudomonas* sp. QS1027 have strong amoebicidal activity (Götze et al., 2023). More information on the influence of predator interactions and predator-derived molecules on LP regulation is needed.

Antiparasitic activities are documented for viscosin against the human parasitic protozoan *Trypanosoma cruzi,* the causal agent of Chagas disease. A viscosin-like LP from *Pseudomonas* sp. H6 was active against the fish parasitic ciliate *Ichthyophthirius multifiliis* and showed inhibitory effects against green algae, crustaceans, cyanobacteria and zebrafish embryos (Raaijmakers et al., 2006; Korbut et al., 2022).

LPs also mediate insect interactions with orfamides, sessilin and viscosin shown to possess insecticidal properties (Geudens and Martins, 2018). Insecticidal activity appears a multifactorial process involving LPs and other metabolites, e.g., Fit toxin, rhizoxin and HCN wherein the role of LPs appears to be strain-specific (Jang et al., 2013; Flury et al., 2017).

### 3.6 Mode of action

The main mode of action of LPs is membrane disruption through pore-formation causing membrane leakage and cell death (Geudens and Martins, 2018). The majority of mechanistic studies rely on model cell membranes that enable simpler and well-controlled experiments however they lack the complexity of real biological membranes. Consequently, the detailed mechanism of action underlying LP pore-formation including membrane selectivity, is largely unknown. As biological organisms are capable of altering their lipid membrane composition in response to external signals in order to adapt to their physical environment (Chwastek et al., 2020), it is likely that LPs have not one but multiple modes of action and display context-dependent activities. This may also contribute to the low resistance towards LPs in the environment despite their ubiquitous nature (Peschel and Sahl, 2006; Steigenberger et al., 2023). Future work should determine the influence of basic membrane parameters, *e.g., membrane thickness* in addition to physical membrane properties and lipid composition of different membrane types, *e.g., bacterial, fungal and mammalian* on LP activity. This was recently highlighted by (Ferrarini et al., 2022a) wherein the bioactivities of tolaasin and sessilin were reduced against oomycetes when membrane sterol composition is altered.

## 4 Biosynthesis

**FIGURE 2:**
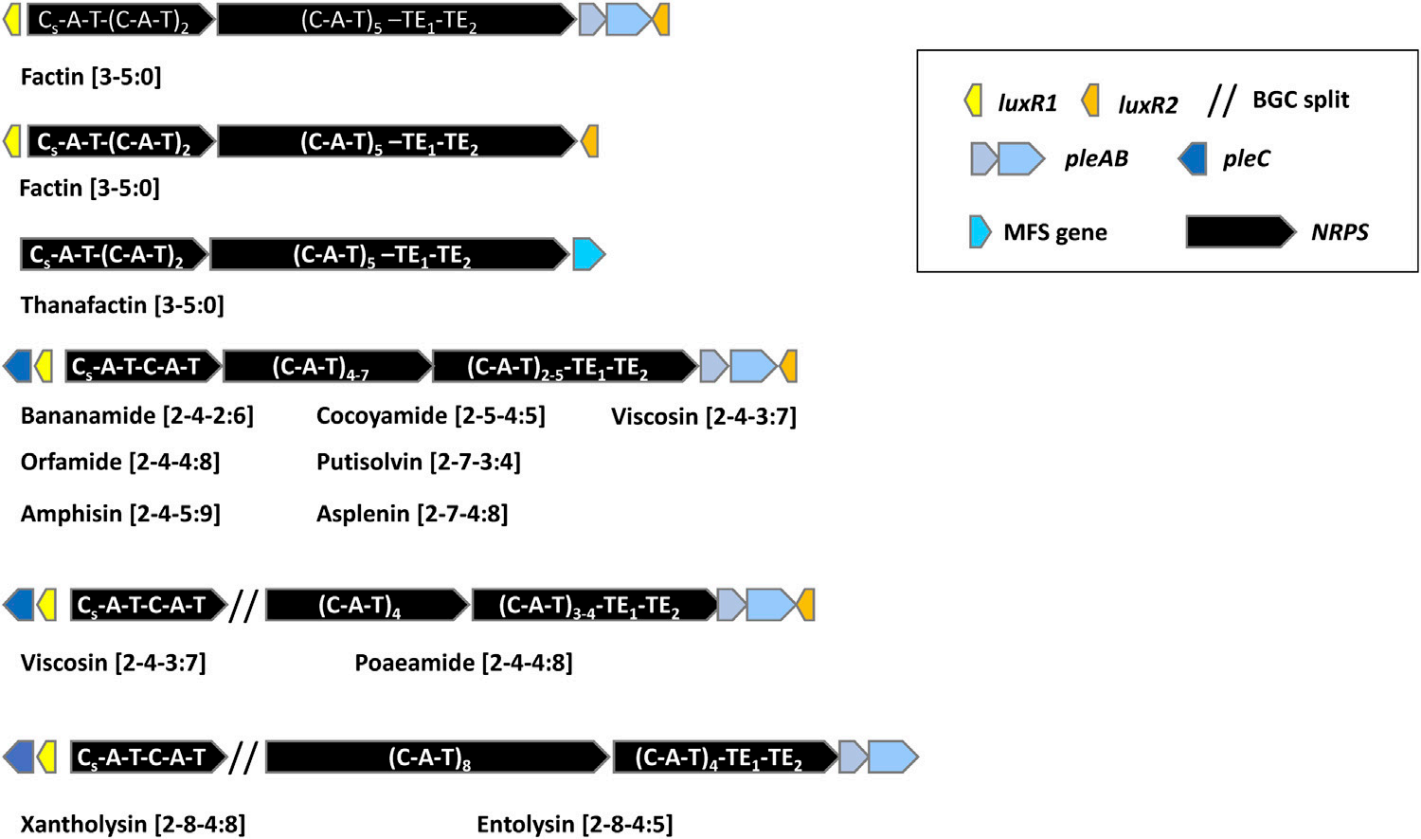
General overview of the organization of lipopeptide biosynthetic gene clusters in lipopeptide mono-producers.

In *Pseudomonas*, LPs are synthesized by large BGCs encoding multi-modular nonribosomal peptide synthetases (NRPSs) (see Götze and Stallforth (2020); Roongsawang et al. (2011) for a detailed overview). These enzymes recognize, activate, modify, and link amino acid intermediates to the product peptide and can synthesize peptides with unusual amino acids including D-amino acids. A typical module comprises a condensation domain, an adenylation domain and a thiolation domain. A specialized condensation starter domain (C_*s*_) with *N*-acylation activity attaches the fatty acid to the first amino acid. The adenylation (A) domain is responsible for amino acid recognition and adenylation, the thiolation (T) domain binds the adenylated amino acid to a phosphopantetheine carrier. A regular condensation (C) domain catalyzes the formation of a peptide bond between two consecutively bound L-amino acids. A condensation domain with built-in epimerization capacity (C/E domain) located downstream of D-amino acid incorporating modules catalyzes the conversion of L-amino acids to D-isomers. Separate epimerization domains as described in NRPSs of *Bacillus* and *Streptomyces* are lacking in *Pseudomonas* NRPSs (Götze and Stallforth, 2020). Cyclization and release of the peptide are carried out by a tandem of thioesterase (TE) domains associated with the last module. The order of modules is usually co-linear to the peptide sequence. CLPs in mono-producers are usually synthesized by NRPS systems encoded by three large open reading frames that are either organized in one operon or are split, with the first NRPS gene typically composed of two modules, located elsewhere in the genome. The NRPS genes are flanked by one or two *luxR-*type regulatory genes and three genes encoding a tripartite PleABC export system. LLPs are synthesized by NRPS systems encoding by two large open reading frames (Figure 2).

**FIGURE 3:**
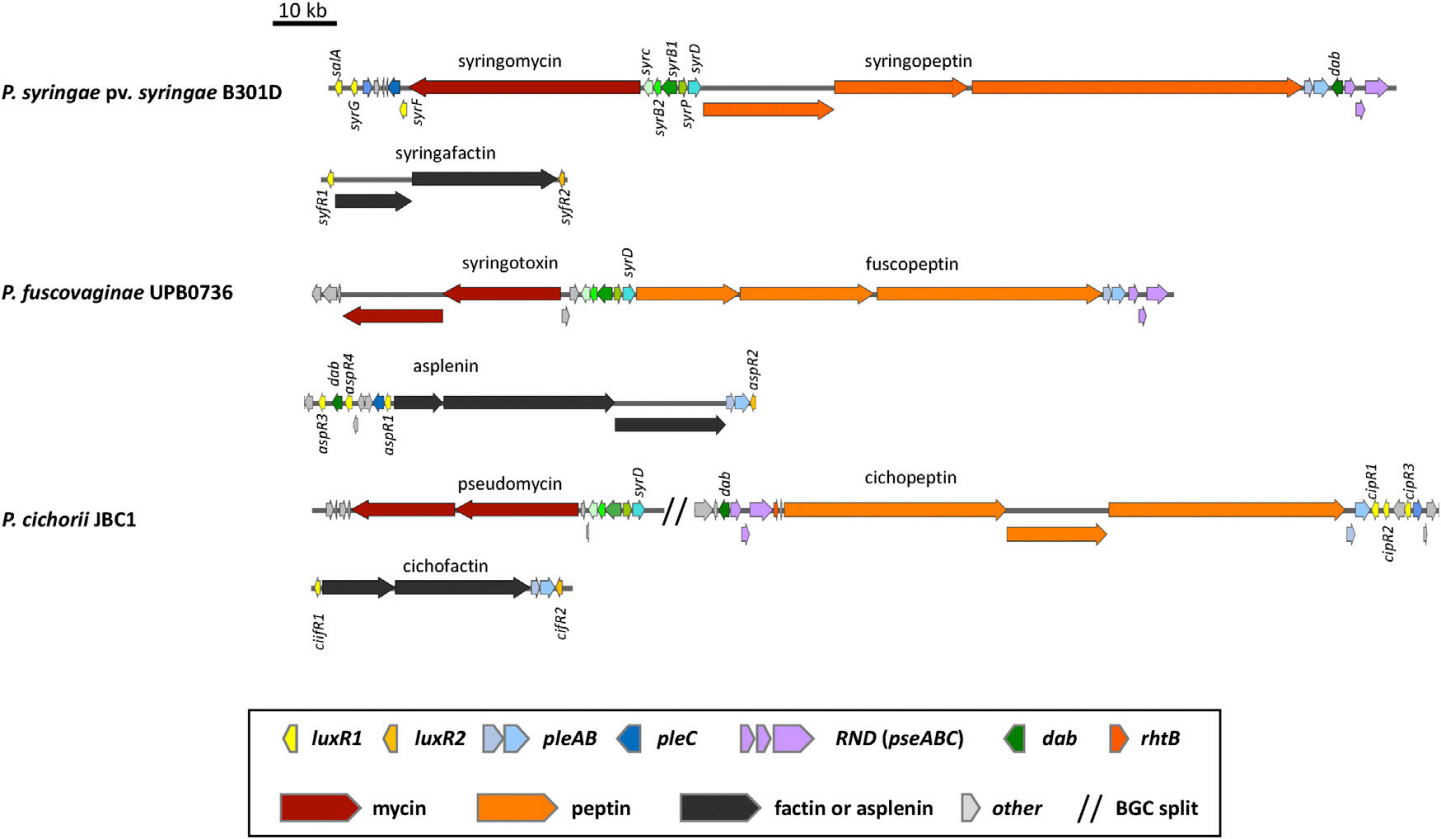
Organization of the lipopeptide biosynthetic gene clusters in non-quorum sensing lipopeptide poly-producers. Representative strains are shown. See Supplementary Table S1 for strain and sequence information.

**FIGURE 4:**
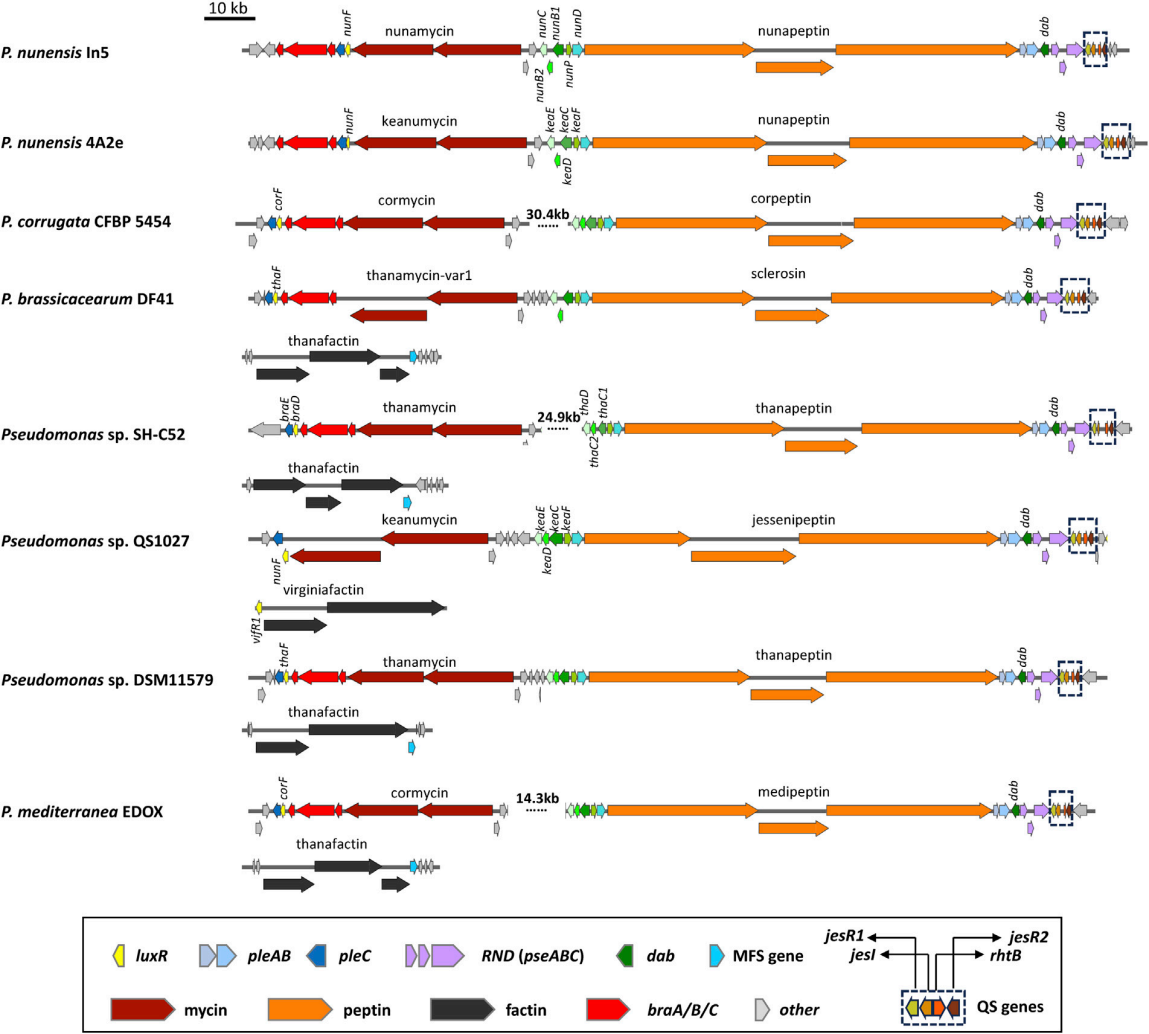
Organization of lipopeptide biosynthetic gene clusters in quorum sensing lipopeptide poly-producers. Representative strains are shown. See Supplementary Table S1 for strain and sequence information.

The chlorinated 9:9 CLPs belonging to the Mycin family are synthesized by one or two NRPSs and separate enzymes encoded by *syrB1*, *syrB2*, *syrC* and *syrP* homologues located upstream of the Mycin BGC (Figures 3, 4). All Mycin family members have the unusual amino acids 3-hydroxy-aspartic acid at position 8 and 4-chlorothreonine at position 9 in the peptide (see Girard et al. (2020) for an overview). SyrB1 is a separate enzyme with an A-T module that activates and loads threonine. SyrB2 is a non-heme Fe^II^ halogenase that chlorinates threonine. SyrC is an aminoacyltransferase that shuttles the threonyl moiety in trans between the T domain of SyrB1 and the T domain of the last module of the NRPS (Bender et al., 1999; Singh et al., 2007). SyrP is an aspartyl hydroxylase that hydroxylates L-Asp after selection, activation and installation of L-Asp on the T domain of the eighth module of the NRPS cluster (Singh et al., 2008). A *pleC* transporter gene is usually located downstream of the Mycin BGC preceded by a *luxR*-type regulatory gene called *syrF* in *P. syringae* pv. *syringae* (Figure 3). CLPs belonging to the Peptin family are usually synthesized by three NRPSs composed of in total 19, 22 or 25 modules. *PleAB* and *pseABC* transporter genes and a *dab* gene are usually located downstream of the Peptin NRPS genes (Figures 3, 4), while A *syrD* transporter gene (Quigley et al., 1993; Quigley and Gross, 1994) is positioned upstream of the Peptin BGC. DAB is involved in the synthesis of 2,4-diaminobutyric acid, a non-protein amino acid present in all peptins and most mycins (Girard et al., 2020). In *Pseudomonas fuscovaginae*, the *dab* gene is located upstream of the asplenin BGC and flanked by two *luxR*-type regulatory genes (Figure 3). In *P. cichorii*, *pleAB* transporter genes and three *luxR*-type regulatory genes are located downstream of the cichopeptin BGC, but *dab* and *pseABC* transporter genes are located upstream of the cichopeptin BGC (Figure 3).

## 5 Regulation

Information on the regulatory mechanisms and environmental signals controlling LPs is lacking but key to modulating LP production both quantitatively and qualitatively in the lab or in the environment for industrial and environmental biotechnology applications. Here, we present what is known so far.

### 5.1 Global regulation of LP-associated genes

**FIGURE 5:**
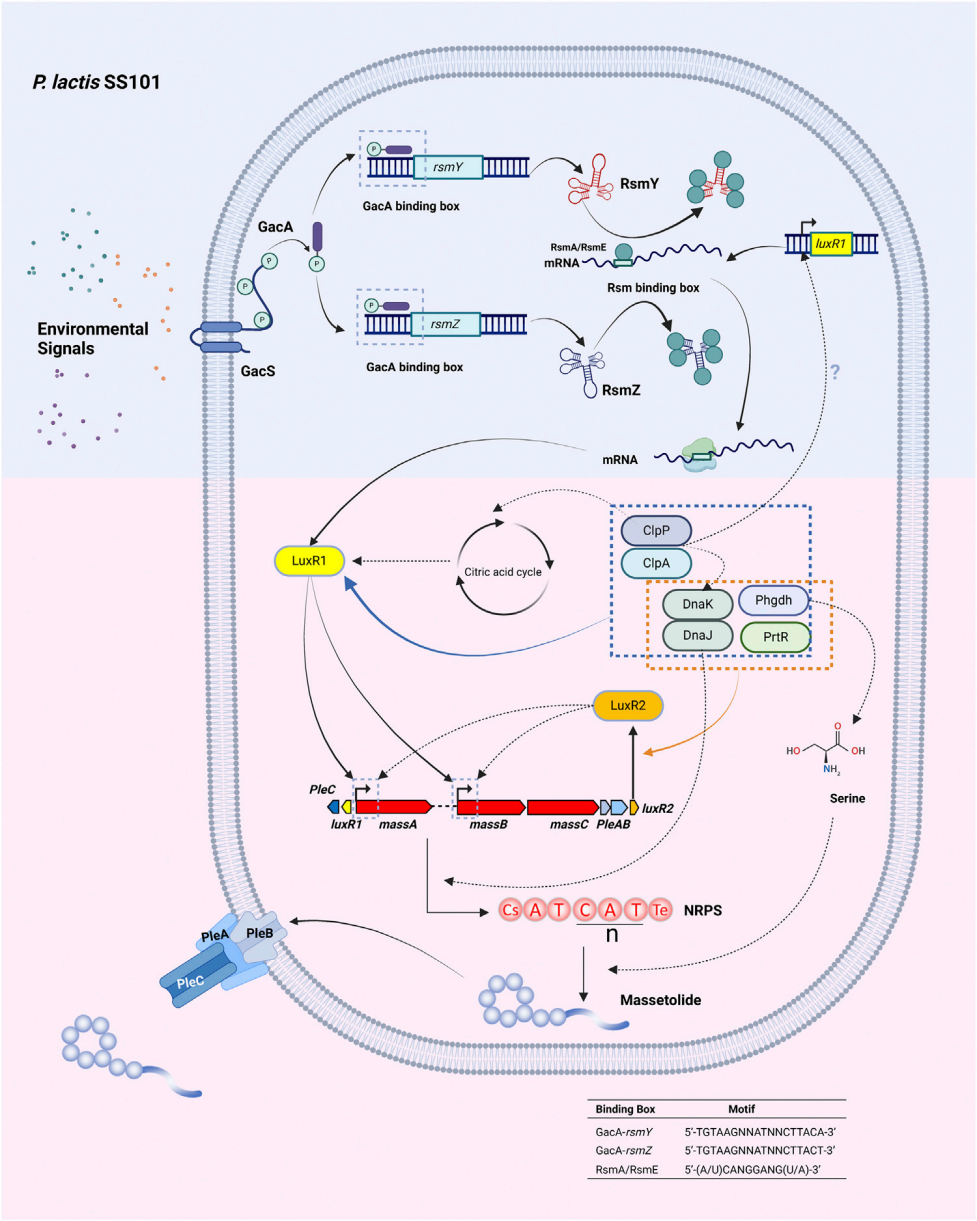
Regulation of lipopeptide production in the massetolide mono-producer *P. lactis* SS101. Massetolide production is under the control of the Gac/Rsm signal transduction pathway. The sensor kinase GacS is triggered by unknown environmental signals in the periplasm resulting in autophosphorylation. The phosphate group is transferred to the GacA response regulator via a phospho-relay system. Phosphorylated GacA binds to the GacA box in the promoter region of the *rsmY* and *rsmZ* genes encoding small RNAs. The resulting small RNAs RsmY and RsmZ bind to the repressor proteins RsmA and RsmE, relieving translational repression at the ribosomal binding site of the mRNAs of *the luxR1 (massAR)* gene. LuxR1 (MassAR) and LuxR2 (MassBCR) activate transcription of the massetolide biosynthetic gene cluster. The serine protease ClpP and its chaperone ClpA additionally regulate massetolide production via LuxR1, the heat shock proteins DnaK and DnaJ, and proteins involved in the citric acid cycle. DnaK and DnaJ may be required for proper folding of LuxR proteins or for assembly of the NRPS complex. Phgdh: D-3 phosphoglycerate dehydrogenase involved in the biosynthesis of the amino acid L-serine. Serine makes up two of the nine amino acids in massetolide. PrtR: antisigma factor which interacts with extra-cytoplasmic function sigma factors and affects the transcription of both *luxR1* and *luxR2* by an unknown mechanism. Secretion of massetolide occurs by the ABC transporter PleABC. Motifs: GacA-binding box see (Humair et al., 2010), Rsm box, see (Olorunleke et al., 2017). Created with BioRender.com.

The best studied global regulatory system associated with LP production is the Gac/Rsm signal transduction pathway (Haas and Défago, 2005) (Figure 5). The cascade is initiated by the GacS/GacA two-component system composed of the membrane-bound GacS sensor kinase and the cognate GacA response regulator located in the cytoplasm. The GacS sensor kinase, initially called LemA, was first described in the bean pathogen *P. syringae* pv. *syringae* strain B728a and found essential to produce the CLP syringomycin (Hrabak and Willis, 1992; Hrabak and Willis, 1993). At high cell densities autophosphorylation of the GacS sensor kinase is triggered by an unknown chemical signal in the periplasm and the phosphate group is then transferred to the GacA response regulator via a phospho-relay mechanism. Phosphorylated GacA triggers the expression of small RNA genes. The resulting small RNAs (rsmX, rsmY, rsmZ) specifically bind to post-transcriptional repressor proteins (RsmA, RsmE) thereby relieving the translation repression exerted by these proteins at the ribosomal binding site of mRNAs encoding genes (Sonnleitner and Haas, 2011) involved in the biosynthesis or regulation of bioactive molecules including LPs. In most LP-producing strains mutations in *gacS* or *gacA* lead to a complete loss of LP production (Koch et al., 2002; de Bruijn et al., 2007; de Bruijn et al., 2008; Song et al., 2014; Olorunleke et al., 2017). Likewise, a *rsmXYZ* mutant in *P. protegens* CHA0 is no longer able to produce the CLP orfamide (Sobrero et al., 2017). Mutations in both *rsmY* and *rsmZ* resulted in loss of massetolide production in *P. lactis* SS101, while a double mutation in *rsmE* and *rsmA* restored massetolide production in a *gacS* mutant. In this strain, the most likely target of the RsmE and RsmA repressor proteins is the LuxR-type regulator MassAR (Song et al., 2015b).

Targeting global regulators is an effective strategy to activate silent gene clusters as demonstrated in several *Streptomyces* studies. In *P. fluorescens* Pf0-1, a silent gene cluster encoding a novel CLP (gacamide) was identified by genome mining and subsequently activated by repairing a defective *gacA* through complementation (Jahanshah et al., 2019).

### 5.2 Regulation of lipopeptide production in mono-producers (Figure 5)

LP mono-producers are found in the *P. fluorescens*, *P. putida* and *P. syringae* group and possess roles in surface motility, biofilm formation or break down, solubilization of nutrients, protection against competitors and predators, induction of systemic resistance in plants, and interactions with insects (D'Aes et al., 2010; Raaijmakers et al., 2010; Götze and Stallforth, 2020; Oni et al., 2022).

**FIGURE 6:**
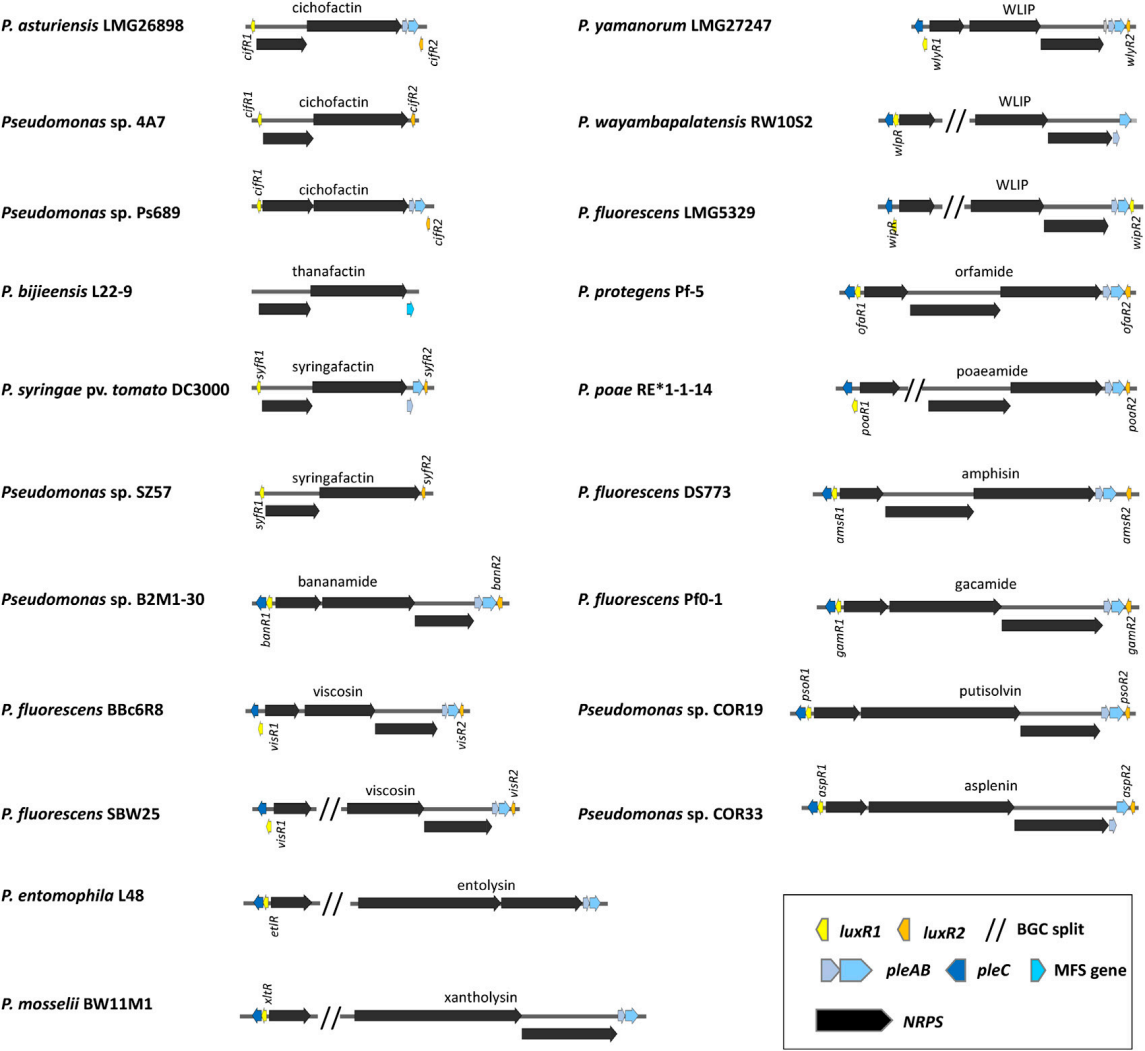
Organization of lipopeptide biosynthetic gene clusters in lipopeptide mono-producers. See Supplementary Table S1 for strain and sequence information.

The organization of the BGCs encoding CLPs in mono-producers is very well conserved with usually three NRPS genes arranged in either one operon, or in a split configuration in which the first NRPS gene is located elsewhere in the genome (Cesa-Luna et al., 2023) (Figure 6).

The genomic regions flanking the NRPS gene clusters encoding CLPs typically contain well conserved regulatory and transporter genes. *Pseudomonas* CLPs are secreted by the PleABC tripartite efflux system, homologous to the MacAB-TolC ABC-type multidrug efflux pump that is found in many Gram-negative bacteria (Fitzpatrick et al., 2017). The PleABC machinery is composed of the inner membrane protein PleB (MacB), the periplasmic adapter PleA (MacA) and the outer membrane protein PleC (TolC) (Girard et al., 2022) (Figure 5). The *pleC* gene is usually located upstream of the first NRPS gene and preceded by a regulatory gene (*luxR1*) encoding a protein of the LuxR family. These LuxR proteins contain a typical DNA-binding helix-turn-helix (HTH) motif but lack an *N*-acylhomoserine lactone (*N*-AHL)-binding domain. The *pleA* and *pleB* genes are positioned downstream of the third NRPS gene and are often, but not always, followed by a *luxR*-type regulatory gene (*luxR2*) transcribed in the opposite direction (Figure 6). Recently is was shown that PleB is suitable as a diagnostic sequence for genome mining allowing the detection and/or typing of *Pseudomonas* LP producers (Girard et al., 2022).

Mutation of either *luxR1* (*viscAR*) or *luxR2* (*viscBCR*) results in loss of viscosin production in *P. fluorescens* strain SBW25 (de Bruijn and Raaijmakers, 2009). A *viscAR* mutation in this strain could be complemented with the *luxR1* (*massAR*) regulatory gene of the massetolide producer *P. lactis (fluorescens)* SS101 (de Bruijn and Raaijmakers, 2009). Mutation of *luxR1* (*arfF*) in *Pseudomonas* sp. MIS38 likewise leads to loss of arthrofactin production (Washio et al., 2010). Mutation of *psoR1* leads to loss of putisolvin production in *P. putida* PCL1445, while mutation of *pleA* (*macA*) or *pleB* (*macB*) in this strain leads to reduced putisolvin production (Dubern et al., 2008). In *P. protegens* CHA0 it has been shown that translation of the LuxR-type regulatory genes *orfR1* and *orfR2* located up- and downstream of the orfamide BGC are under the direct control of the Gac/Rsm pathway (Sobrero et al., 2017).

LuxR2-type regulatory proteins are lacking in BGCs encoding xantholysin (Li et al., 2013) and entolysin (Vallet-Gely et al., 2010), while the situation is variable for WLIP producers (Figure 6). A *luxR2* gene is present downstream of the WLIP BGC in *P. yamanorum* LMG27247 (*wlyR2*), *P. fluorescens* LMG5329 (*wipR2*) and two WLIP producers from the *P. putida* group (*P. xanthosomae* COR54 (*wlfR2*) and *P. fakonensis* COW40 (*wlfR2*)), but absent in *P. wayambapalatensis* RW10S2 (Rokni-Zadeh et al., 2012) (Figure 6). The LuxR1 regulators XltR, EltR, WlpR and WipR are required for xantholysin, entolysin and WLIP production in the respective strains *P. mosselii* BW11M1, *Pseudomonas entomophila* L48, *P. wayambapalatensis* RW10S2, and *P. fluorescens* LMG5329 (Vallet-Gely et al., 2010; Rokni-Zadeh et al., 2012; Li et al., 2013; Rokni-Zadeh et al., 2013). Intriguingly, all LP BGCs that lack *luxR2* are produced by strains that taxonomically belong to the *P. putida* group (Girard et al., 2021). The reason for this is unknown but could symbolize a different ecological role.

Mono-producers of the LLPs syringafactin and cichofactin are described in various phylogroups of the *P. syringae* group (Bricout et al., 2022; Cesa-Luna et al., 2023) and in some isolates of the *P. putida group* (Cesa-Luna et al., 2023). LLPs are encoded by two NRPS genes arranged in an operon. Homologues of *luxR1* and *luxR2* are present upstream and downstream of the BGC encoding syringafactin in *P. syringae* DC3000, and cichofactin in *P. putida* 4A7, but the *pleC* transporter gene is absent in *P. syringae* DC3000 (Berti et al., 2007) and no transporter genes are present in the BGC encoding cichofactin in *P. putida* 4A7 (Figure 6). Mutation of *luxR1* (also called *syfR*), but not of *luxR2* (pspto2833) leads to loss of syringafactin production in *P. syringae* DC3000 (Berti et al., 2007). *P. bijieensis* L22-9 is a thanafactin mono-producer. The thanafactin BGC lacks regulatory genes, and a major facilitator superfamily (MFS) transporter is located downstream of the BGC (Figure 6).

**FIGURE 7:**
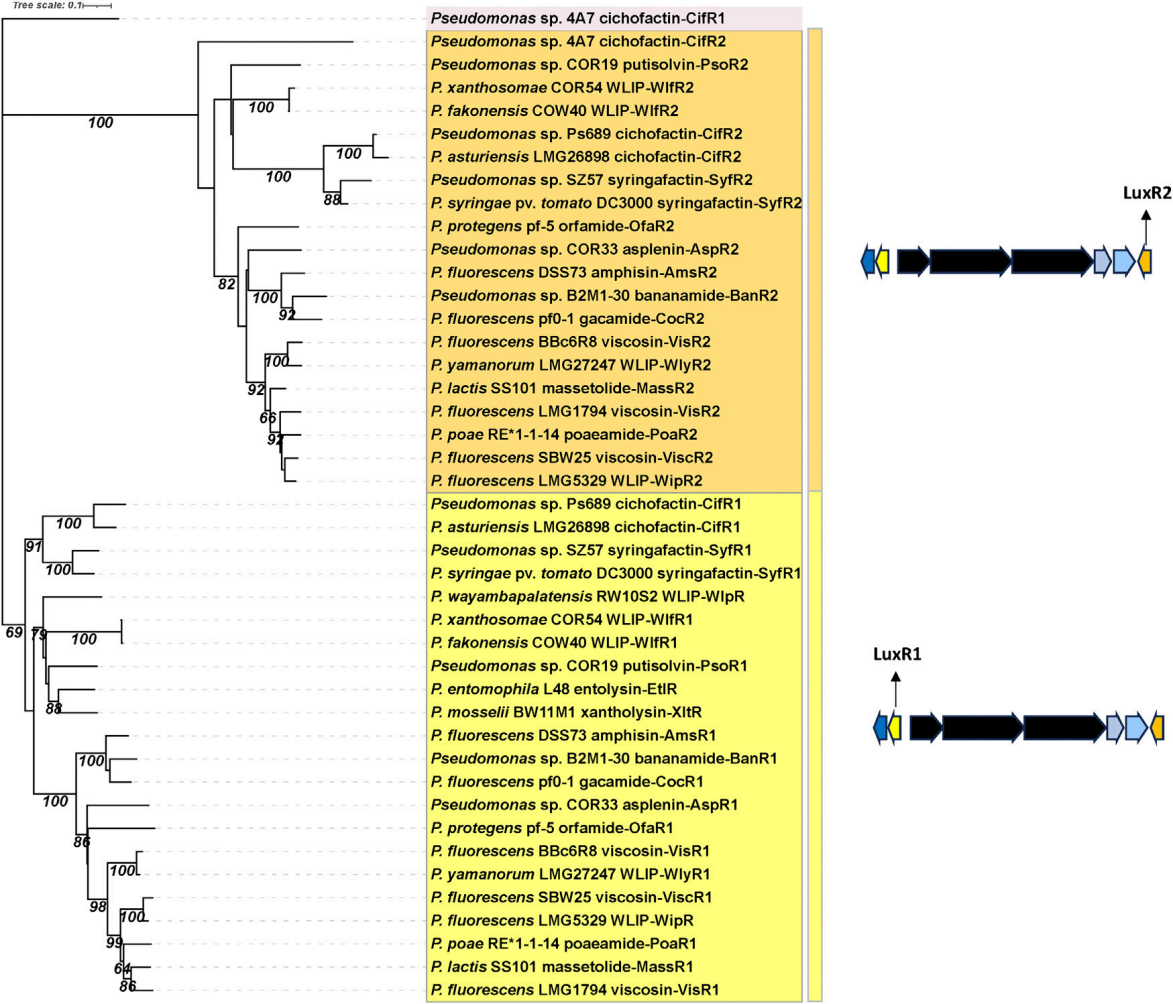
Phylogenetic tree of LuxR-type regulatory proteins in lipopeptide mono-producers. Neighbor-Joining phylogenetic tree (JTT model) constructed with MEGA 11 with MUSCLE alignment of LuxR amino acid sequences from LP mono-producers. Bootstrap values (percentage of 1,000 replicates) are shown in the figure. LuxR1 protein encoding genes (yellow) are located upstream of the lipopeptide NRPS genes, LuxR2 protein encoding genes (orange) are located downstream of the lipopeptide NRPS genes. See Supplementary Table S1 for strain and sequence information.

Phylogenetic analysis shows that LuxR1 and LuxR2 proteins from CLP and LLP mono-producers form two distinct phylogenetic groups (Figure 7).

Regulation of LP production in mono-producers in response to cell density by autoinducers, also called quorum sensing regulation (Venturi, 2006), has to our knowledge only been described for the viscosin producer *P. fluorescens* 5064 (Cui et al., 2005) and in the putisolvin producer *P. putida* PCL1445. *P. fluorescens* 5064, an opportunistic soft rot pathogen of broccoli, produces the 9:7 CLP viscosin that is important for plant surface colonization. The N-acyl homoserine lactone (HSL) quorum sensing signal 3-OH-C_8_-HSL regulates viscosin production in this strain (Cui et al., 2005). *P. putida* PCL1445 was isolated from grass roots grown in soil polluted with polyaromatic hydrocarbons. The strain produces the 12:4 CLPs putisolvin I and II. These compounds inhibit biofilm formation and break down existing *Pseudomonas* biofilms (Kuiper et al., 2004). The *ppuI-rsaL-ppuR* quorum sensing system is involved in putisolvin production and mutants impaired in either *ppuI* or *ppuR* show a severe reduction in putisolvin production (Dubern et al., 2006). The quorum-sensing signals 3-oxo-C_10_-*N*-acyl homoserine lactone (3-oxo-C_10_-AHL) or 3-oxo-C_12_-AHL induce expression of the biosynthesis genes activating production of putisolvin I and II in this strain (Dubern et al., 2006).

Additional compounds involved in LP regulation in mono-producers include heat shock proteins, Clp proteases and enzymes involved in amino acid metabolism (Figure 5). The Hsp70 class heat shock protein DnaK regulates putisolvin production together with DnaJ at low temperature in *P. putida* PCL1445 (Dubern et al., 2005). DnaK is also involved in the regulation of massetolide in *P. lactis* SS101 (Song et al., 2014). Mutation of the gene encoding the Hsp90 class heat shock protein HtpG leads to loss of arthrofactin synthesis, while arthrofactin biosynthesis genes are normally expressed suggesting a role in posttranscriptional processes (Washio et al., 2010). Heat shock proteins may be required for the proper folding of positive (LuxR-type?) transcription factors or for assembly of the NRPS complex (Song et al., 2014). In *P. putida*, *DnaK* is under the control of the Gacs/GacA regulatory system, but this may not be the case in *P. lactis* SS101. In *P. lactis* SS101 the serine protease ClpP and its chaperone ClpA are required for massetolide biosynthesis. ClpP is an ATP-dependent serine protease that associates with different ATPases, including ClpA. ClpA selects target proteins for degradation by ClpP. Transcriptomic and proteomic analyses suggest that the ClpAP complex regulates massetolide biosynthesis via the LuxR1 transcriptional regulator MassAR, the heat shock proteins DnaK and DnaJ and via proteins involved in the citric acid cycle (Song et al., 2015a) (Figure 5). Additional regulators identified in *P. lactis* SS101 include D-3-phosphoglycerate dehydrogenase (Phgdh) and the antisigma factor PrtR (Song et al., 2014). Phgdh is involved in the biosynthesis of L-serine, an amino acid that makes up two of the nine amino acids in massetolide. PrtR interacts with extra-cytoplasmic function sigma factors of the sigma 70 family and regulates the expression of *luxR1* and *luxR2* by an unknown mechanism (Figure 5).

Concerning environmental signals that regulate LP production in mono-producers Mazzola et al. (2009) showed that the massetolide and viscosin biosynthesis genes, *massABC* and *viscABC* respectively*,* in *P. lactis* SS101 and *P. fluorescens* SBW25 are upregulated upon protozoal exposure conferring protection to each strain against predation. Interestingly, the authors observed that physical contact between prey and predator was not necessary to activate the *massABC* and *viscABC* genes. It would be interesting to test extracts from different protists in order to identify specific protist signals that trigger CLP production.

So, for LP mono-producers, quorum sensing regulation is not common, the LuxR1 regulator is always present (with the notable exception of the thanafactin producer *P. bijieensis* L22-9) and essential for LP production, while LuxR2 is sometimes lacking and when present, not always essential for LP production. LuxR1 and in some cases also LuxR2 are under translational control of the Gac/Rsm regulon. LP production is additionally regulated by heat shock proteins and the ClpAP complex.

### 5.3 Regulation of lipopeptide production in poly-producers (Figure 8)

LP poly-producers belonging to the *P. syringae* group, or *P. corrugata* and *P. mandelii* subgroup within the *P. fluorescens* group, typically co-produce CLPs from the Peptin and Mycin family and often produce an additional LLP of the Factin family, while poly-producers of the *P. asplenii* subgroup produce an additional CLP of the Asplenin family (Girard et al., 2020). Strains that produce a CLP of the Tolaasin family belonging to the *P. fluorescens* subgroup co-produce a CLP of the Viscosin family, while some members of the *P. protegens* group co-produce sessilin (a member of the tolaasin family) and orfamide (Cesa-Luna et al., 2023). Regulation of LP production in poly-producers is more complex than in mono-producers and can be quorum sensing dependent or independent.

#### 5.3.1 Non-quorum sensing regulatory systems in Mycin and Peptin producers

Production of Mycins and Peptins is independent from QS in various plant pathogenic *Pseudomonas* bacteria including the closely related bean pathogens *P. syringae* pv. *syringae* B301D and B728a, the wide host range pathogens *P. cichorii* JBC1 and SF1-54, and the rice pathogen *P. fuscovaginae* UPB0736. These plant pathogenic *Pseudomonas* strains produce two CLPs simultaneously, one representative from the Peptin family, and one from the Mycin family. These CLPs always seem to co-occur (Figure 3). They function as phytotoxins, are usually co-produced and their secretion involves the same transporters. They can act synergistically to cause disease on plants by forming pores in plant membranes (Bender et al., 1999). Plant pathogenic CLP-producing *Pseudomonas* strains usually attack above-ground plant parts such as leaves or leaf sheaths. The third LLP produced by plant pathogens such as *P. syringae* pv. *syringae*, *P. cichorii* and *P. fuscovaginae* is not directly involved in virulence, but is essential for swarming motility and *in planta* colonization (Girard et al., 2020).

**FIGURE 8:**
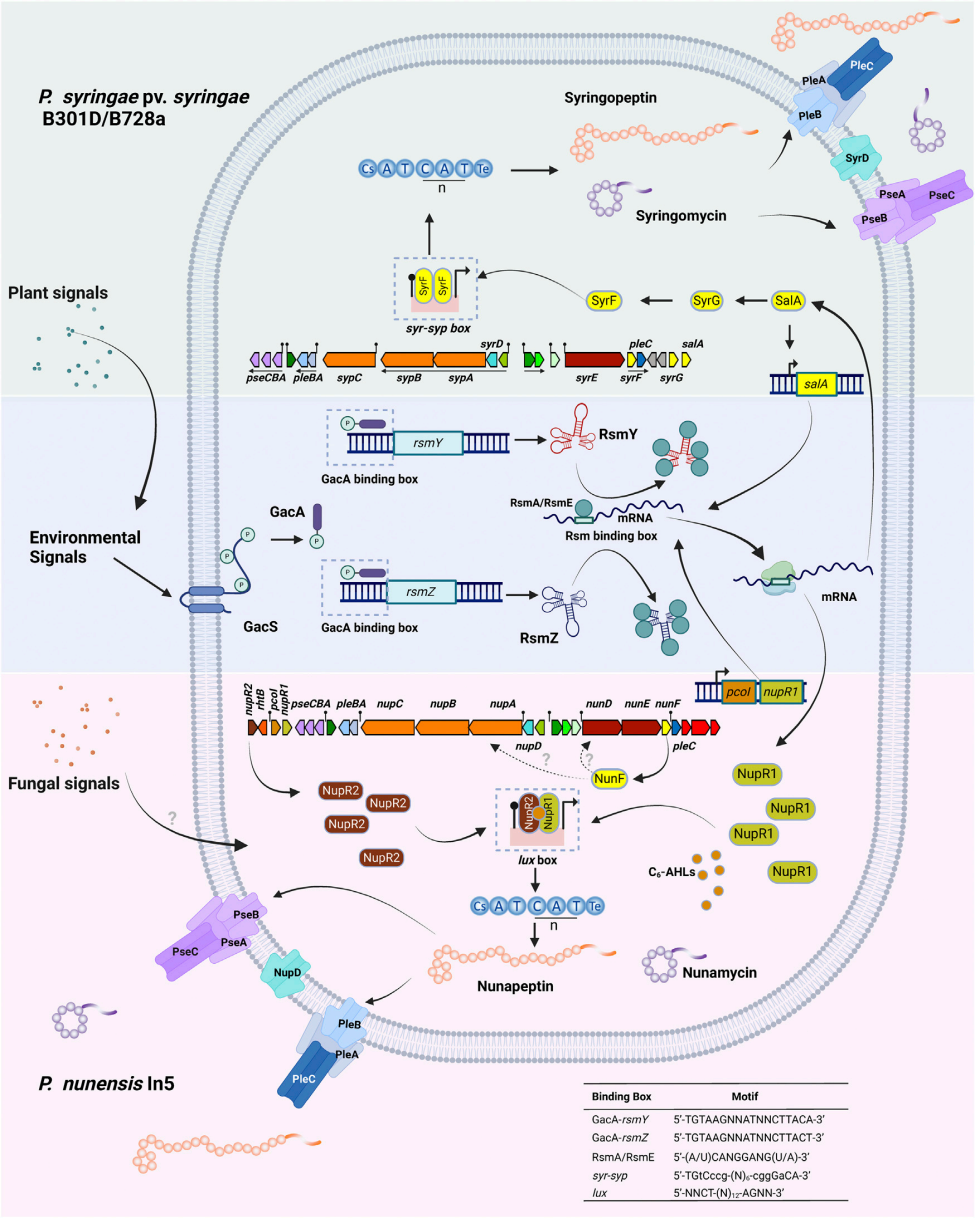
Regulation of lipopeptide production in Mycin and Peptin producers. **Top:** Non-quorum sensing regulation of lipopeptide production in the syringomycin and syringopeptin producers *P. syringae* pv. *syringae* B301D and B728a. Syringomycin and Syringopeptin production is under the control of the SalA regulon. SalA is activated by the Gac/Rsm signal transduction pathway in response to plant signals. SalA positively regulates its own transcription and activates the expression of both *syrG* and *syrF.* SyrG acts as a transcriptional activator of *syrF*. SyrF binds as a homodimer to a specific *syr-syp* box (indicated with a pin) in the promoter region of syringomycin and syringopeptin biosynthesis genes and transporters. Secretion of syringomycin and syringopeptin occurs by the ABC transporter PleABC, the cytoplasmic membrane protein SyrD and the RND transporter PseABC. Operons in the biosynthetic gene cluster are underlined. **Bottom:** Proposed model of quorum-sensing regulation of lipopeptide production in the nunamycin and nunapeptin producer *P. nunensis* In5. PcoI is an acyl-homoserine lactone (AHL) synthase encoding the autoinducer N-hexanoyl-L-homoserine lactone (C_6_-AHL), NupR1 is a LuxR family protein lacking an N-AHL binding domain under the control of the Gac/Rsm regulon, NupR2 is a LuxR protein with an N-AHL binding domain. The promoter region of nunamycin and nunapeptin biosynthesis genes and transporters harbor a specific *lux* box (indicated with a pin) to which a NupR1-NupR2-C_6_-AHL complex may bind (not experimentally proven). Production of nunamycin and nunapeptin is triggered by fungal signals that activate *nunF.* How NunF further activates lipopeptide synthesis and secretion is unknown. Nunamycin and nunapeptin secretion probably occurs by the ABC transporter PleABC, the cytoplasmic membrane protein NupD and the RND transporter PseABC. Motifs: GacA-binding box, see (Humair et al., 2010); Rsm-binding box, see (Olorunleke et al., 2017); *syr-syp* box, see (Wang et al., 2006a); *lux-*box, see (Whiteley and Greenberg, 2001). Created with BioRender.com.

Most of our current knowledge on CLP regulation stems from the phytotoxins syringomycin and syringopeptin produced by *P. syringae* pv. *syringae.* Production of these compounds is dependent on the global regulatory GacS/GacA system in addition to the LuxR-type transcription factors SalA, SyrF, and SyrG which combined activate CLP synthesis in response to plant signal molecules. In *P. syringae* pv. *syringae* B301D and B728a, *syrF, salA and syrG* are associated with the syringomycin BGC (Figure 8) (Lu et al., 2002). The resulting proteins all carry a C-terminal HTH DNA binding motif typical for LuxR regulatory genes, but lack an *N*-terminal AHL domain and belong to the fourth subfamily of the LuxR superfamily (Wang et al., 2006a; Vaughn and Gross, 2016). SalA, SyrF and SyrG were shown to control syringopeptin and syringomycin biosynthesis in a hierarchical organization (Lu et al., 2005; Vaughn and Gross, 2016). The *salA* gene is positively regulated by the GacS/GacA regulon. SalA positively regulates its own expression (Kitten et al., 1998) but also the expression of both *syrG* and *syrF* (Lu et al., 2002; Wang et al., 2006a)*,* while SyrG functions as an transcriptional activator of *syrF* (Vaughn and Gross, 2016). SyrF activates the syringomycin and syringopeptin biosynthesis and transporter genes by binding to a specific *syr-syp* box in the promoter region as a dimer (Figure 8). Plants signals that trigger the production of syringomycin and syringopeptin include the phenolic glycoside arbutin and sugars that occur in large quantities in leaf tissues such as D-fructose. GacS, SalA and SyrF transduce the plant signals to activate the syringomycin and syringopeptin BGCs. Sensing of the plant signal molecules probably occurs via GacS (Wang et al., 2006b).

*P. syringae* pv. *syringae* strains produce a third 8:0 LLP called syringafactin that is needed for swarming motility and enhances fitness on leaf surfaces by attracting moisture and facilitating access to nutrients (Burch et al., 2014). Two *luxR1* and *luxR2* type regulatory genes named *syfR1* and *syfR2* are situated up and downstream of the syringafactin BGC in *P. syringae* pv. *syringae* strains (Figure 3). Expression of both *syfR1* and the surfactin biosynthesis gene syfA are dependent on SalA in *P. syringae* pv. *syringae* B728a (Hockett et al., 2013). No *pleAB*-type transporter genes are associated with the syringafactin BGC in *P. syringae* pv. *syringae* B301D (Figure 3) and B728a in contrast to the syringafactin BGC in the mono-producer *P. syringae* DC3000 (Figure 6).

**FIGURE 9:**
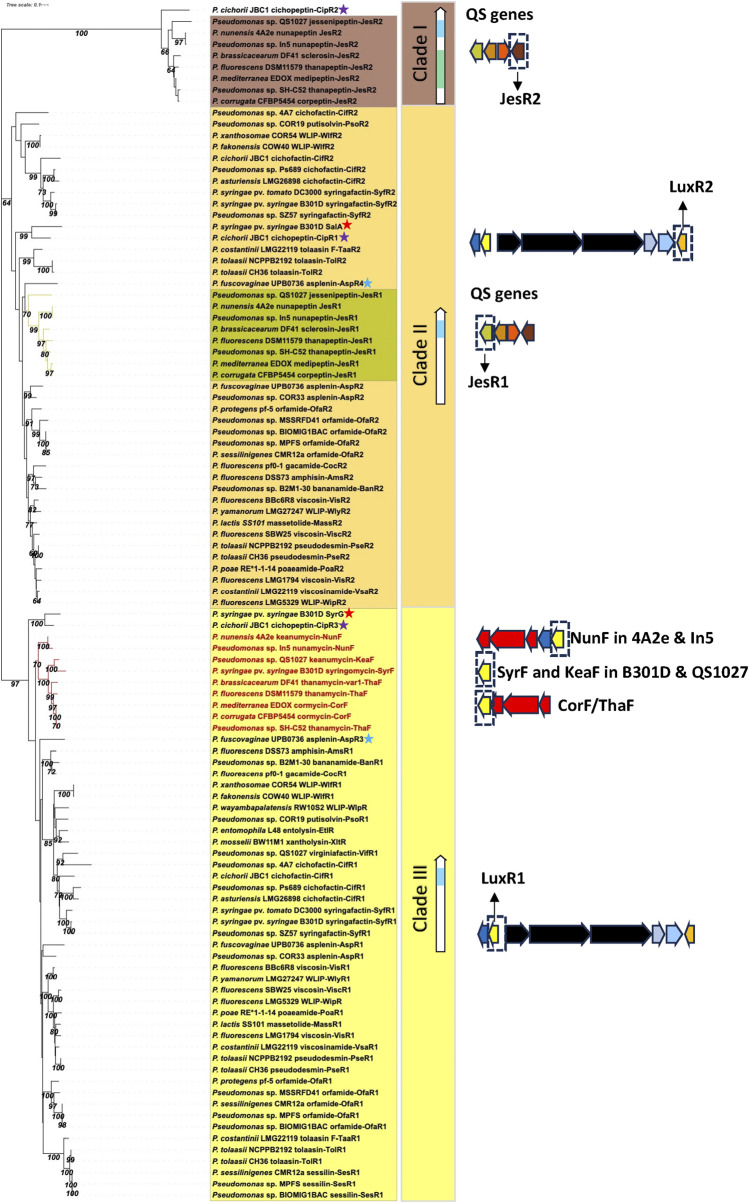
Phylogenetic tree of LuxR-type regulatory proteins in lipopeptide mono- and poly-producers. Neighbor-Joining phylogenetic tree (JTT model) constructed with MEGA 11 with MUSCLE alignment of LuxR amino acid sequences from mono- and poly-producers. Bootstrap values (percentage of 1,000 replicates) are shown in the figure. Clade I: JesR2-type regulatory proteins with a helix-turn-helix (HTH, indicated in blue) and an autoinducer binding motif (indicated in green) produced by LP poly-producers. This clade also contains the CipR2 protein from *P. cichorii* (purple star). Clade II: LuxR2-type regulatory proteins with a HTH motif (indicated in blue) found in LP mono-producers downstream of the LP BGCs. This clade also contains the JesR1 type regulators found in poly-producers with a quorum sensing system (indicated in green) and the SalA (red star), CipR1 (purple star) and AspR4 (blue star) regulatory proteins of *P. syringae* pv. *syringae*, *P. cichorii* and *P. fuscovaginae*, respectively. Clade III: LuxR1-type regulatory proteins with a HTH motif (in blue) located upstream of the LP BGCs in mono- and poly-producers. This clade also contains the SyrF homologues (indicated in red in the tree) located downstream of the Mycin or Brabantamide BGCs in LP poly-producers. See Supplementary Table S1 for strain and sequence information.

The rice sheath brown rot pathogen *P. fuscovaginae* UPB0736 belongs to the *P. asplenii* subgroup and produces the 19:5 CLP fuscopeptin, the 9:9 CLP syringotoxin and the 13:8 CLP asplenin. Syringotoxin and fuscopeptin act synergistically in inhibiting plant H^+^-ATPase activity in plant membranes (Batoko et al., 1998) and both CLPs are involved in causing sheath rot symptoms on rice. In addition, syringotoxin is also toxic to the rice sheath blight pathogen *Rhizoctonia solani* AG1-1A. Asplenin is needed for swarming motility (Ferrarini et al., 2022b). The syringotoxin and fuscopeptin BGCs in *P. fuscovaginae* UPB0736 are completely devoid of *luxR* type regulatory genes, while three *luxR* genes (termed *luxR1*, *luxR2* and *luxR3* by (Ferrarini et al., 2022b) and renamed here as *asp3*, *asp4* and *asp1*) are situated upstream of the asplenin BGC, and one *luxR* gene (*aspR2*) downstream of the last NRPS gene of this cluster (Figure 3). Phylogenetic analysis reveals that AspR3 and AspR1 cluster in the same clade as the LuxR1 regulators situated upstream of the BGCs in mono-producers, while AspR2 and AspR4 cluster with the LuxR2 regulators downstream of the BGCs in mono-producers (Figure 9). Intriguingly, aspR1 and aspR2 genes are flanking the asplenin BGC in the asplenin mono-producer *Pseudomonas* sp. COR33 (Figure 6), but this strain lacks the *aspR3* and *aspR4* genes. The function of the LuxR regulators in *P. fuscovaginae* is unknown but it is likely that they co-regulate all three CLPs.

*P. cichorii* is a broad-host range pathogen that produces the 22:8 CLPs cichopeptin A and B (Huang et al., 2015), the 8:0 LLPs cichofactin A and B (Pauwelyn et al., 2013) and a third 8:8 Mycin-type CLP that is presumably pseudomycin (Girard et al., 2020). Cichopeptins are important virulence factors in the lettuce midrib rot pathogen *P. cichorii* SF1-54 and cause necrotic symptoms on leaves (Huang et al., 2015). Cichopeptins contain two residues of glycine in their peptide backbone and production is stimulated by glycine betaine. Cichopeptins are produced *in planta* at early stages of infection (Huang et al., 2015). Cichofactins are needed for swarming motility and a cichofactin-mutant formed significantly more biofilm. They are produced *in planta* and needed for *in planta* spread of *P. cichorii* but are not phytotoxic *per se* (Pauwelyn et al., 2013). In *P. cichorii* JBC1 and SF1-54 three *luxR*-type regulatory genes (*cipR1, cipR2, cipR3*) are located downstream of the cichopeptin BGC and two *luxR*-type regulatory genes (*cifR1, cifR2*) up and downstream of the cichofactin BGC (Figure 3). The cichofactin BGC contains *pleAB* transporter genes, but *pleC* is lacking. CipR1 associated with the cichopeptin BGC cluster is similar to SalA (about 60% identity) from *P. syringae* pv. *syringae* and clusters in clade II, while CipR3 is similar to SyrG (about 70% identity) and clusters in clade III in a phylogenetic tree in which all LuxR-type regulatory proteins associated with LP BGCs are included (Figure 9). Intriguingly, CipR2 carries an N-AHL binding domain (see further), but the genome of JBC1 does not encode LuxI type proteins involved in AHL synthesis. The role of these regulatory proteins in LP production in *P. cichorii* has to our knowledge not been studied.

#### 5.3.2 Quorum sensing regulatory systems in Mycin and Peptin producers

Strains producing Mycin and Peptin variants that are quorum sensing regulated are found within the *P. asplenii*, *P. mandelii* and *P. corrugata* subgroups of the *P. fluorescens* group or complex (Girard et al., 2020). These strains produce a 19:5 or 22:5 CLP of the Peptin family, a second CLP of the Mycin family that is composed of two NRPSs, and often a third LLP of the Factin family. Many of these strains also harbor a brabantamide BGC downstream of the Mycin cluster. Brabantamides are cyclocarbamate antibiotics with activity against Gram-positive bacteria and Oomycetes (Van Der Voort et al., 2015). Brabantamide genes are co-transcribed with the Mycin and Peptin BGCs and possibly also co-secreted with Mycins and Peptins (Girard et al., 2020). The Mycin, brabantamide and Peptin BGCs are located on a so-called pathogenicity island, designed LPQ (lipopeptide/quorum sensing) island because conserved quorum sensing genes are located downstream of the peptin cluster in all these strains (Melnyk et al., 2019) (Figure 4).

*P. nunensis* In5 produces the 22:5 CLP nunapeptin and the 9:9 CLP nunamycin but is not known to produce a third LLP (Figure 4; Figure 8). Studies in *P. nunensis* In5 reported an inter-kingdom communication cascade that upon detection of fungal signals activates a *Pseudomonas* specific regulator called NunF required for expression of the antifungal CLPs nunamycin and nunapeptin. A *nunF* mutant is unable to produce nunamycin and nunapeptin (Hennessy et al., 2017a). The *nunF* promoter showed no induction with plant signal molecules, but was induced during co-culture with *Fusarium graminearum*, and by unknown components of a *Fusarium*-derived fungal extract in addition to pure fungal-associated molecules trehalose and glycerol (Hennessy et al., 2017b; Christiansen et al., 2020). NunF is a homologue of SyrF in the plant pathogen *P. syringae* pv. *syringae*. The *syrF* promoter however, showed a lower induction by fungal extract and hardly any induction by trehalose (Christiansen et al., 2020).

Most CLP poly-producers belonging to the *P. corrugata*, *P. asplenii* and *P. mandelii* subgroup carry a homologue of *syrF/nunF* downstream of the Mycin BGC or Mycin/brabantamide BGCs, followed by a *pleC* transporter gene (Figure 4) such as *nunF* associated with nunamycin in *P. nunensis* In5, and keanumycin in *P. nunensis* 4A2e and *Pseudomonas* sp. QS1027, *corF* associated with cormycin/brabantamide in *P. corrugata* CFBP5454 and *P. mediterranea* EDOX, and *thaF* (*braD*) associated with thanamycin/brabantamide in *Pseudomonas* sp. SH-C52. A *syrF* homologue is also present downstream of the uncharacterized Mycin in *P. brassicacearum* DF41 (Figure 4). Given that all these strains show strong antifungal activity, it is likely that their NunF/SyrF homologues also react to fungal signals, but this remains to be investigated. The NunF/SyrF-type regulatory proteins form a separate cluster in clade III (indicated in red in Figure 9), a clade that also contains all LuxR1-type regulators located upstream of LP BGCs in mono-producers.

Some strains also encode a third LLP that is either thanafactin or virginiafactin (Figure 4). The thanafactin BGCs lack *luxR*-type regulatory genes and all contain an MFS transporter downstream of the BGC that it typical for thanafactin producers. The virginiafactin BGC in *Pseudomonas* sp. QS1027 lacks transporters but carries a *luxR1*-type regulatory gene (*vifR1*) upstream of the first NRPS gene. The VifR1 protein clusters with LuxR1-type proteins located upstream of cichofactin and syringafactin in mono- and poly-producers in clade III (Figure 9).

In addition, all these strains harbour a four-gene quorum sensing system composed of *jesR1 (rfiA, nupR1)*, *jesI (pcoI, pdfI)*, *rhtB (orf1)* and *jesR2 (pcoR, nupR2, pdfR)* downstream of the Peptin BGC and preceded by an operon (*pcoABC*) encoding an RND transporter system (Figure 4). The PcoABC efflux system is homologous to the PseABC RND efflux system in *P. syringae* pv. *syringae* B301D involved in the secretion of syringomycin and syringopeptin (Kang and Gross, 2005). JesI (PcoI, PdfI) is an AHL synthase, JesR2 (PcoR, NupR2, pdfR) is a LuxR family protein with an *N*-terminus AHL-binding domain, while JesR1 (RfiA, NupR1) is a LuxR family protein with a helix-turn-helix motif but lacking an *N*-AHL binding domain. In *P. corrugata* CFBP 5454 mutants in *pcoR*, *rfiA,* or *pcoI* and *rfiA* are unable to produce and/or secrete cormycin and corpeptin (Licciardello et al., 2009). In *Pseudomonas* sp. QS1027 production of jessenipeptin is regulated by a QS system involving the AHL signal hexanoyl homoserine lactone (C6-AHL). Interestingly, the biosynthesis genes for jessenipeptin are located adjacent to those encoding another specialized metabolite mupirocin that works in synergy with the CLP against *methicillin-resistant S. aureus* (MRSA). However, the AHL signal required to induce production of the CLPs differ. Deletion of *jesI*, *jesR1* or *jesR2* led to a complete suppression of jessenipeptin production (Arp et al., 2018). Pflanze et al. (2023) have shown that the regulatory network governing production of CLPs in *P. nunensis* 4A2 required for protection against predation by amoeba and nematodes involves LuxR-type regulatory genes and the QS signal N-hexanoyl-L-homoserine lactone (C_6_-AHL). *PcoI*, *nupR1* or *nupR2* mutants in *P. nunensis* 4A2 no longer produce keanumycin or nunapeptin and the authors were able to demonstrate that complementation of knockout mutants with the signaling molecule C_6_-AHL restored CLP production (Pflanze et al., 2023). C_6_-AHL also regulates CLP production in other strains and has been detected in chemical extracts of *P. nunensis* In5 when nunamycin and nunapeptin are produced (Hennessy et al., 2017a). The situation is slightly different in *P. brassicacearum* DF41 where an AHL deficient strain expressing the AHL lactonase gene *aiiA* from *Bacillus subtilis* still produced sclerosin, but the *rfiA (jesR1)* mutant was strongly reduced in sclerosin production (Berry et al., 2014). In this strain *pdfI* and *rfiA* are co-transcribed and positively regulated by the Gac-Rsm network. Recently, it was shown that a QS system is also involved in the regulation of medpeptin by *P. mediterranea* S58 however the specific AHL signal required is unknown (Gu et al., 2023). A model showing how quorum sensing may regulate Mycin and Peptin production is depicted in Figure 8.

Phylogenetic analysis reveals that the JesR2 homologues in the various strains cluster together in a separate subgroup (clade I) that also contains the CipR2 protein with an N-AHL binding domain associated with the cichopeptin BGC in *P. cichorii* JBC1. JesR1 homologues form a distinct subgroup (indicated in green) within clade II harboring all LuxR2 proteins associated with LP mono-producers (Figure 9).

#### 5.3.3 Regulatory systems in tolaasin producers

Tolaasin is the main virulence factor of the mushroom pathogens *P. tolaasii* and *P. costantinii* (Scherlach et al., 2013). *P. tolaasii* can occur in two reversible phenotypic variants, the pathogenic or smooth phenotype that is opaque, mucoid, non-fluorescent and produces tolaasin, and a non-pathogenic or rough variant that is translucent, non-mucoid, fluorescent and no longer produces tolaasin. Switching between the two phenotypes occurs by a reversible duplication of a 661 bp element in the 5’ end of a regulatory gene called *pheN* (Grewal et al., 1995) or *rtpA* (Murata et al., 1998), but that is actually the homologue of *gacS* (Heeb and Haas, 2001). The duplication introduces a frameshift mutation that results in the loss of part of the sensor domain of GacS (PheN) (Han et al., 1997). Compounds of the *P. ostreatus* fruiting body activate tolaasin production. Nonpathogenic variants occur at 22°C–30°C but not at 17°C and 20°C or in the presence of *Pleurotus* extracts (Murata et al., 1998).

**FIGURE 10:**
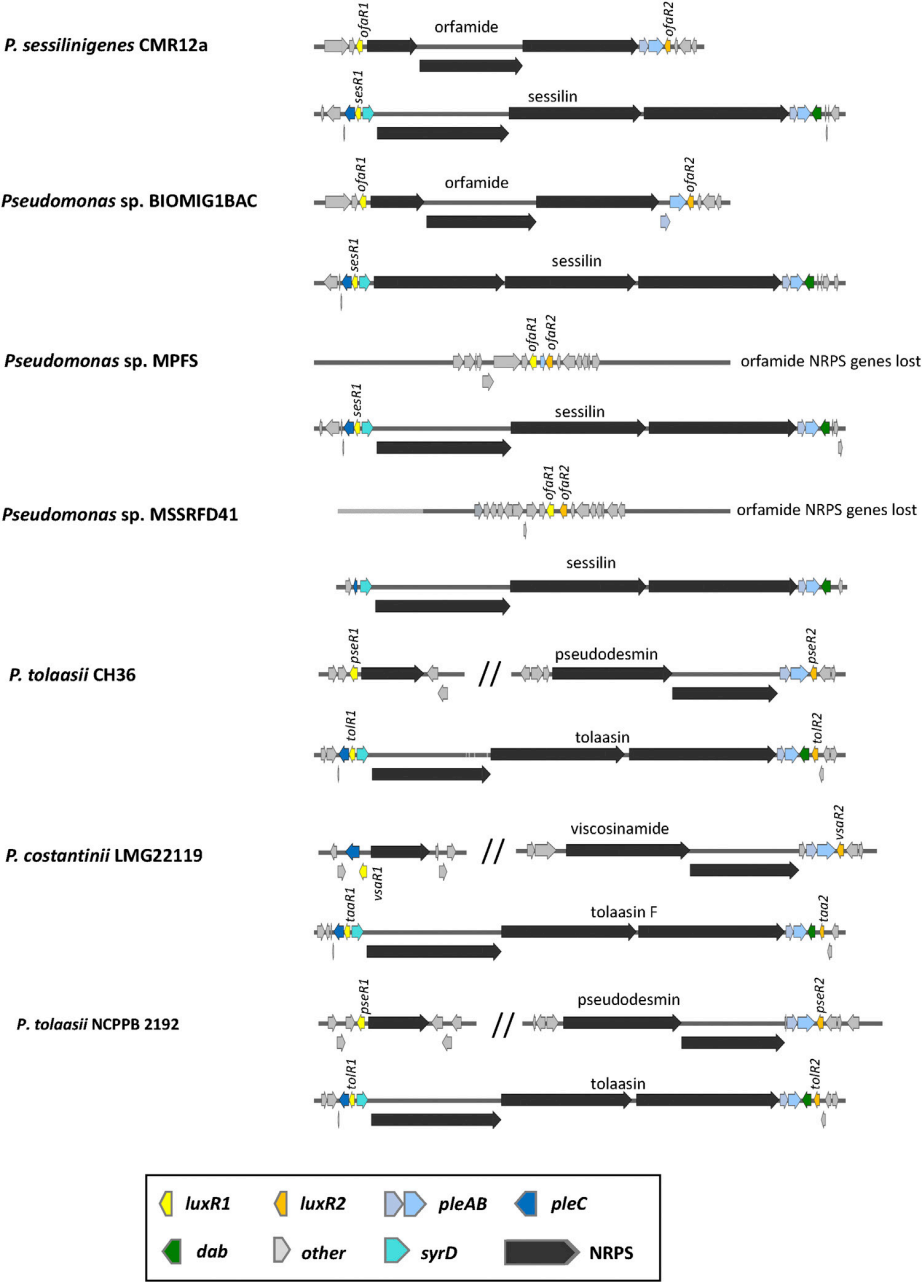
Organisation of lipopeptide biosynthetic gene clusters in tolaasin/sessilin producers. See Supplementary Table S1 for strain and sequence information.

*P. tolaasii* and *P. costantinii* also produce a second CLP of the viscosin family (pseudodesmin or viscosinamide), required to colonize the mushroom cap (Hermenau et al., 2020; Cesa-Luna et al., 2023). In the tolaasin/pseudodesmin producers *P. tolaasii* NCPBB 2192^T^ and CH36, and the tolaasin/viscosinamide producer *P. costantinii* LMG22119 (Cesa-Luna et al., 2023) *luxR1* and *luxR2*-type regulatory genes (*tolR1/taaR1* and *tolR1/taaR2*) are present up and downstream of the tolaasin BGC next to the transporter genes *pleC* and *pleAB*. Similar regulators are also present in the split second BGCs encoding pseudodesmin and viscosinamide, respectively. Unlike pseudomodesmin mono-producers, however (Oni et al., 2020a), the pseudodesmin BGCs lack a *pleC* type transporter (Figure 10).

A variant of tolaasin, called sessilin, is produced by the well-studied biocontrol strain *P. sessilinigenes* CMR12a, which also produces the 10:8 CLP orfamide (D’aes et al., 2014). Sessilin/orfamide co-producers have also been obtained from an urban wastewater treatment plant in Turkey (*Pseudomonas* sp. BIOMIG1BAC) (Altinbag et al., 2020) and from the rhizosphere of banana in Sri Lanka (*Pseudomonas aestus* BW16M1) (Cesa-Luna et al., 2023). The taxonomically closely related strains *Pseudomonas* sp. MPFS isolated from the skin of a treefrog in Brazil (Brunetti et al., 2022) and *Pseudomonas* sp. MSSRFD41 from the rhizosphere of finger millet in India (Sekar et al., 2018) only produce sessilin (Cesa-Luna et al., 2023) (Figure 10). The BGC for sessilin in *P. sessilinigenes* CMR12a is located on a genomic island acquired by horizontal gene transfer that also contains a phenazine BGC (Biessy et al., 2019).

In *P. sessilinigenes* CMR12a, typical *luxR1* and *luxR2*-type regulatory genes (*ofaR1* and *ofaR2*) are present up and downstream of the orfamide BGC (Figure 10), like in orfamide mono-producers such as *P. protegens* CHA0 and Pf-5 (Ma et al., 2016a). The orfamide BGC in CMR12a lacks a *pleC* transporter gene (Olorunleke et al., 2017), while this gene is present in orfamide mono-producers (Ma et al., 2016a). Only one LuxR-type regulatory gene (*sesR1*) is found upstream of the sessilin BGC next to a *pleC* transporter gene in strain CMR12a (Figure 10). Functional analysis of LuxR type regulators in CMR12a has revealed that *ofaR1* and *ofaR2* mutants are completely abolished in both orfamide and sessilin production, while a *sesR1* mutant is still able to produce the two CLPs and has no clear phenotype. Rsm binding sites are located upstream of all three *luxR*-like genes suggesting regulation by the Gac/Rsm system (Olorunleke et al., 2017). In *P. sessilinigenes*, phenotypic switching by duplication of a fragment in the *gacS* gene is not known to happen. Intriguingly, however, spontaneous variants of *P. sessilinigenes* strains that have lost the genomic island with the sessilin and phenazine BGC occur both in the lab and on plants (Omoboye, 2019). The sessilin/orfamide co-producers *Pseudomonas* sp. BIOMIG1B and *P. aestus* BW16M1 have a very similar sessilin and orfamide BGC organization as CMR12a (Figure 10). *Pseudomonas* sp. MPFS and *Pseudomonas* sp. MSSRFD41 also carry a sessilin BGC, but strain MSSRFD41 has lost the *luxR1-type* regulatory gene *sesR1* and part of the *pleC* transporter. Intriguingly, both strains have lost the orfamide BGC, which is considered part of the core genome of *P. protegens* but retained the *ofaR1* and *ofaR2* regulatory genes associated with the orfamide BGC in CMR12a and BIOMIG1B (Figure 10). Moreover, strain MPFS still has part of the *pleB* transporter again suggesting a gene loss.

Phylogenetic analysis reveals that OfaR1/VsaR1/PseR1 and SesR1/TolR1/TaaR1 cluster with other LuxR1 regulators located upstream of LP BGCs in clade III, while OfaR2/VsaR1/PseR2 and TolR2/TaaR2 clusters with the LuxR2 regulators located downstream of the LP BGCs in mono-producers in clade II (Figure 9).

It remains to be investigated whether the LuxR regulators associated with the pseudodesmin/viscosinamide BGC co-regulate tolaasin production in *P. tolaasii* and *P. costantinii* and whether the PleAB transporters downstream of the tolaasin and viscosinamide/pseudodesmin BGCs share the outer membrane protein PleC_tol_ for CLP secretion.

## 6 Production

As highlighted above, LPs are multifunctional molecules with boundless *potential* applications in research and industry. However, high production cost and low titers are major bottlenecks in their commercialization. Information on the specific conditions that favor LP production in *Pseudomonas* is scarce and the factors limiting their production largely unknown. Here, we describe LP production in natural *versus* controlled lab-scale environments and present strategies to modulate production *in vitro* and/or *in situ.*

### 6.1 LP production in natural environments

Owing to the complexity of natural environments, a significant proportion of the *Pseudomonas-*LP research performed to date is only based on lab-based analyses. Production of several CLPs in the environment notably amphisin, tensin and viscosinamide has been detected, for example, in the sugar beet rhizosphere (Nielsen and Sorensen, 2003). Interestingly in this study no LPs were found in bulk soil signifying production is niche specific (Nielsen and Sorensen, 2003). Production of LPs by other isolates was also quantified on beet seeds and found in ranges of 0.22–0.65 µg CLP per seed. The *P. fluorescens* strain DSS73 (originally isolated from the sugar beet rhizosphere) was shown to produce amphisin on germinating sugar beet seeds in soil correlating with lab-based findings that unknown components of a sugar beet extract induce *amsY* expression needed for amphisin production (Koch et al., 2002). The environmental conditions required for amphisin production thus appear to reflect the producing strain’s specific habitat. A similar observation was made for *P. syringae* pv *syringae* where conditions required for lab-based production of syringomycin reflect the environmental conditions required for pathogenesis (Gross, 1985). As amphisin has antifungal properties it would be interesting to determine whether components of fungal extracts also induce *amsY* expression. Interestingly, the concentration of LPs detected over time remained similar in sterile soil whereas levels in non-sterile soil were rapidly reduced suggesting degradation by indigenous microbes (Nielsen and Sorensen, 2003).

While numerous studies have indicated that LPs are susceptible to degradation in the environment, the detailed mechanisms underpinning this process remain obscure. However, a key outcome of such research to date is the finding that microbial degradation of LPs can result in structural changes that alter their biological activities. For example, in *Bacillus*, degradation of surfactin generates a linear surfactin which can no longer induce systemic resistance (ISR) in tobacco (Rigolet et al., 2023). Linear surfactant also displays a reduced ability to lower surface tension when compared to cyclic surfactin (Liu et al., 2015). In the common button mushroom, protective helper bacteria disarm the causal agent of brown blotch *P. tolaasii* by enzymatic linearization of the toxin tolaasin and surfactant pseudodesmin, required to colonize the mushroom cap, to yield inactive linear forms of the CLPs (Hermenau et al., 2020). The linearization of CLPs is thought to be a resistance mechanism against competing bacterial species (Hermenau et al., 2020). More recently (Hansen et al., 2023), reported the cooperative degradation of orfamide A by *Rhodococcus globerulus* D757 and *Stenotrophomonas indicatrix* D763 to protect orfamide-sensitive members of a synthetic community during co-culture with the orfamide-producer *P. protegens*. It has been proposed that degradation of CLPs could be a strategy deployed by competitors to prevent CLP producers from performing “critical” functions in their environment such as biofilm formation, enhancing motility or colonization of specific niches (Rigolet et al., 2023). In another recent study (Zhang et al., 2021), present a new role of LPs in mediating bacterial cooperation to evade amoebal predation. Synthesis of syringafactins by *Pseudomonas* sp. SZ57 induces peptidase production in *Paenibacillus* sp. SZ31 resulting in partial LP-degradation yielding a mix of modified natural products that become amoebicidal (Zhang et al., 2021). These findings expand current knowledge on the ecological functions of LPs and demonstrate how interactions with other microbes can be exploited to unlock production of new compounds. Additionally, the identification of specific compounds capable of natural product modification could be exploited to select for the synthesis of LPs with specific functions during, for example, lab or large-scale cultivations (see Section 6.4).

Going forward it will be critical to quantify production of LPs *in situ* and correlate expression of LP-associated genes to LP production. Moreover, information on the stability of LPs in the environment, the mechanisms by which they are degraded and importantly the impact of degradation on LP structure and function is key for developing applications in environmental biotechnology and potentially opens a new avenues for natural product discovery.

### 6.2 LP production in controlled lab-scale environments

LP production is influenced by growth phase in addition to abiotic factors (e.g., temperature, pH, oxygen) and nutritional factors (e.g., carbon, nitrogen and phosphorus sources, trace elements) (Raaijmakers et al., 2006). However, comparable studies are limited and the information available is scattered across a handful of strains (Nybroe and Sorensen, 2004). Optimization of growth conditions required for LP production is best described for syringomycin in *P. syringae* (Gross and DeVay, 1977; Gross and DeVay, 1977; Gross, 1985; Grgurina et al., 1996) showed higher levels of syringomycin production in still potato dextrose broth cultures (PDB) compared to aerated cultures. Viscosinamide is also produced in still cultures or under carbon, nitrogen or phosphorus starvation (Nybroe and Sorensen, 2004). Whereas syringomycin and syringopeptin are produced in the stationary phase, production of viscosinamide, tensin and amphisin is growth-coupled and occurs in the exponential phase (Nielsen et al., 1999; Nielsen et al., 2000; Koch et al., 2002). Syringomycin is regulated by iron (>2 µM), requires L-histidine as a nitrogen source and is repressed by inorganic phosphate (Gross, 1985). In plant tissues, levels of iron are high whereas concentrations of inorganic phosphate are not sufficient to inhibit the phytotoxin demonstrating that the environmental conditions required to support syringomycin production correlate with those necessary for pathogenesis (Gross, 1985).

While pH had no effect on syringomycin production, temperature was identified as an important factor with optimal production recorded at 24°C (Gross, 1985). In *P. nunensis* In5, regulation of nunamycin and nunapeptin is also temperature-dependent with optimal production at 15°C. Antifungal activity of In5 decreases with increasing temperature correlating with a reduction in production of both LPs (Michelsen and Stougaard, 2011; Christiansen et al., 2020). Production of putisolvin in *P. putida* PCL1445 is likewise temperature dependent with the highest production at 11 °C (Dubern et al., 2005). Also, syringafactin production in *P. syringae* pv. *syringae* is thermoregulated with much higher production at 20°C than at 30°C (Hockett et al., 2013). The molecular mechanisms underpinning the effects of temperature on CLP production are not well known. It has been suggested that temperature may directly impact synthetase formation and thereby alter iron uptake required for specialized metabolism (Gross, 1985). For *P. putida* PCL1445 it was shown that low temperature positively regulates putisolvin production during the late exponential phase via the DnaK stress response system (Dubern et al., 2005).

Screening for cultivation conditions inducing nunamycin and nunapeptin production revealed that nunapeptin is produced on a range of media both liquid and agar-based personal communication. In contrast, nunamycin production occurs on select agar-based media or in defined liquid minimal media supplemented with either glucose, glycerol and trehalose as carbon source (Christiansen et al., 2020). Glycerol also supports viscosin production by *P. antarctica* and has been used as a carbon source for rhamnolipid production (Zhao et al., 2021; Ciurko et al., 2023). Carbon source has been shown to influence tensin production in *P. fluorescens*, phytotoxin production in *P. syringae* and fungitoxin production in *P. nunensis* In5 (Nielsen et al., 2000; Woo et al., 2002; Christiansen et al., 2020). In contrast to nunapeptin, production of nunamycin appears to be tightly regulated occurring only under select conditions at low quantities. Nunamycin is a potent antimicrobial peptide and therefore potentially toxic to In5. Interestingly, production of syringomycin which displays a similar structure to nunamycin is not toxic to *P. syringae* at concentrations naturally produced (Gross, 1985). Gross (1985) observed that high syringomycin titers did not negatively impact bacterial growth as comparable cell densities were recorded for producers and non-producers. Finally, culture optimization has also been reported for thanamycin production in *P. fluorescens* SH-C52 resulting in a 3.3-fold product increase sufficient to recover 40 mg/120 L culture for NMR studies however the specific parameters altered were not specified (Johnston et al., 2015).

### 6.3 Strategies to improve LP production

One major obstacle in natural product research is activating silent BGCs and/or improving the production of those expressed at low levels. For *Pseudomonas* LPs the challenge typically resides in low titers rather than no production. Several approaches can be used to activate or enhance BGC expression including cultivation-based approaches, molecular based techniques or synthetic biology strategies and combinatorial chemistry (Reen et al., 2015).

Cultivation-based approaches involve optimization of cultivation conditions, *e.g., nutrients, carbon source, aeration, pH, temperature* or using environmental cues, e.g., *chemicals* to induce compound production. While some information on optimal conditions needed for LP production exist, more systematic screening of culture conditions and in particular the identification of specific environmental signals that positively (or negatively) regulate expression of LP-associated genes is needed. In the well-studied and prolific producers of SMs actinomycetes, a number of key triggers have been identified to access antibiotic production including chemicals, microbial metabolites, interactions with microbes, environmental factors and enzymes that could serve as a useful starting point for studies in *Pseudomonas* (Seyedsayamdost, 2014; Zhu et al., 2014; Rosen and Seyedsayamdost, 2017; Zong et al., 2022). Information on the regulatory pathways and environmental signals influencing LPs can then be integrated into cultivation-based approaches and depending on the application, combined with other strategies, *e.g., strain engineering* to further enhance LP production.

*Pseudomonas* spp. are becoming increasingly attractive cell factories for the production of high-value chemicals including native and non-native SMs in part due to their capacity to utilize cheap carbon sources (Nikel et al., 2014; Wang et al., 2020). Using renewable resources, e.g., agricultural and food waste would greatly increase the commercialization potential of CLPs making bioprocessing of these compounds more sustainable (Ceresa et al., 2023). Importantly, *Pseudomonas* spp. are naturally competent and typically well-suited to genetics and molecular research. When selecting strains (particularly environmental isolates) for improved LP production it will be necessary to determine their genetic tractability and consider their origin, e.g., *beneficial or pathogen*. Select strains can then be engineered using traditional techniques, e.g., *homologous recombination* or next-generation CRISPR-Cas9 based technologies for increased production of target LPs either by introducing genes that promote LP production, deleting genes that inhibit LP biosynthesis, or overexpressing genes using native or synthetic promoters (Batianis et al., 2020). Depending on the complexity of the regulation, simple single-gene mutations to more complex multi-gene knockouts and/or insertions may be required.

*In Pseudomonas* LP-producers, genetic manipulation of regulatory genes has been limited to functional genomics studies and not used to improve LP titers. Interestingly, a study investigating the regulation of massetolide and viscosin demonstrated that the *massA* gene encoding the first NRPS needed for massetolide production can be heterologously expressed to complement a *viscA* mutant deficient in the first NRPS needed for viscosin production (de Bruijn and Raaijmakers, 2009). However, for the LuxR-type regulators only *massAR* (*luxR1*) and not *massBCR* (*luxR2*) could restore viscosin production in the *viscAR* (*luxR1*) mutant (de Bruijn and Raaijmakers, 2009) indicating that while some LP genes can be exchanged among different *Pseudomonas* strains, differences in the functionality of structural and regulatory genes may be at play.

Metabolic engineering can be used to increase LP production using strong indigenous or artificial promoters to increase the copy number of biosynthesis and/or regulator genes or alternatively using inducible promoters to overexpress entire gene clusters. For example, the use of promoter systems to improve LP production has been successful in the rhamnolipid producing strains *P. aeruginosa*, *Burkholderia kururiensis* and *P. chlororaphis* (Chong and Li, 2017). Overexpression of the *rhlAB* operon required for rhamnolipid biosynthesis under the *tac* promoter in *B. kururiensis* yielded a mixture of over 50 rhamnolipid congeners (Tavares et al., 2013). Similarly, overexpression of the rhamnolipid biosynthesis gene *rhlC* in *P. chlororaphis* resulted in the synthesis of di-rhamnolipids instead of mono-rhamnolipids (Solaiman et al., 2015). These findings demonstrate the potential of metabolic engineering-based approaches not only to increase product titer but equally to alter the structure and function of LPs.

Synthetic biology can serve to overcome challenges in engineering native strains for large scale production, for example, by refactoring BGCs to reduce the complexity of LP regulation (Temme et al., 2012; Wang et al., 2021). However, cloning and heterologous expression of large NRPS gene clusters that can span over 100 Kb (Meleshko et al., 2019) is difficult. Drawbacks include PCR amplification or synthesis of large DNA fragments, decrease in cloning efficiency, stability of vectors and/or successful integration onto the chromosome. Moreover, compared to *Pseudomonas* spp., classical hosts such as *E. coli* and yeast are not natural producers of LPs and may, depending on the LP encounter toxicity issues. Furthermore, with the rapid development of advanced gene engineering techniques, e. g., *CRISPRi toolbox* precise editing of model and natural *Pseudomonas* genomes for the controllable manipulation of gene expression will greatly facilitate strain engineering strategies for improved production in native hosts or other *Pseudomonas* strains (Batianis et al., 2020; Wirth et al., 2020).

For pharmaceutical or health-related applications where high purity grade compounds are needed it may be more desirable to synthesize LPs *in vitro*. While total chemical synthesis of the *Pseudomonas* CLPs from the Viscosin, Bananamide and Entolysin family has been achieved, the synthesis of larger LP molecules, for example, found in the poly-producers is more challenging (De Vleeschouwer et al., 2014; De Vleeschouwer et al., 2016; De Roo et al., 2022; Ji et al., 2023; Muangkaew et al., 2023). Chemical synthesis is also used to increase the chemical diversity of molecules, for structure-function studies, and to elucidate the stereochemistry of CLPs (Steigenberger et al., 2021; De Roo et al., 2022; Muangkaew et al., 2023).

### 6.4 Developing a bioprocess for LP production

Limited studies on optimization of the conditions necessary to induce synthesis and improve production of LPs exist. Consequently, no *Pseudomonas*-derived LPs are currently commercially available with the exception of rhamnolipids used in broad-spectrum applications and produced at industrially viable yields (Soberón-Chávez et al., 2021).

Bioreactor-scale production of LPs has only been reported for pseudofactins (PFs), 8:6 CLPs from the Bananamide family in *P. fluorescens* BD5 (Biniarz et al., 2018; Biniarz et al., 2020). Optimal production of PFs requires high glycerol (80 g/L) and tryptone (15 g/L) concentrations with high culture aeration (30 L/min) to achieve 7.2 g/30 L yield of PFs (Biniarz et al., 2018). LPs are often produced as a mixture of LPs comprising variants with minor structural changes that can greatly impact their bioactivity (Dufour et al., 2005; Eeman et al., 2006; Biniarz et al., 2020). Biniarz et al. (2018) observed that media supplementation with valine and leucine causes a shift in the ratio of PFs produced and can be used to select structural LP variants. The selective production of LPs will greatly benefit the purification of LPs for structure-function studies and potentially provide opportunities to identify new LP variants with novel biological activities.

Going forward it will be important to determine the limiting factors of LP production across scales (lab-bioreactor-technical scale). Additional parameters influencing growth and metabolic activity to optimize for include temperature, pH, oxygen, agitation, speed or vessel type (Guez et al., 2021). More basic studies on media and culture conditions required for LP production are needed to (i) routinely produce LPs of interest at lab scale for biological and chemical characterization, and (ii) to scale production from lab-to-bioreactor to develop an efficient bioprocess for LP production. Moreover (Gross and DeVay, 1977), observed that syringomycin production varied considerably across different *P. syringae* strains. Thus, it will be important to determine the specific impact of growth phase, carbon source, nutrients and inducer molecules on LP production in individual strains and to tailor culture conditions accordingly.

## 7 Conclusion and future perspectives

LPs are clearly attractive molecules with enormous potential for versatile and eco-friendly applications within biotechnology. However, low titers coupled with high production costs continue to constrain their commercial development.

Targeting key regulators of LP pathways to improve production in *Pseudomonas* is a promising yet underexplored avenue. To achieve this, future *Pseudomonas*-LP research efforts should focus on (i) understanding the regulatory mechanisms controlling LP production and integrate knowledge on the influence of environmental signals; (ii) strain engineering for improved production and (iii) media optimization and fermentation conditions for scalable manufacturing.

Moreover, it is becoming increasingly evident that LPs are pivotal to the ecological fitness of *Pseudomonads* in the environment. Additional information on why and when LPs are produced in natural environments to facilitate versatile *Pseudomonas* lifestyles in diverse habitats will provide new insights into the ecological functions of these molecules and potentially open new avenues for natural product discovery.

Ultimately, expanding our understanding of the regulatory mechanisms and environmental signals influencing the biosynthesis, structure and function of LPs is key to developing and optimizing industrial-scale production and wide-spread use of these “green compounds” for the future.

## References

[B1] Abdel-Mawgoud A. M. Lépine F. Déziel E. (2010). Rhamnolipids: diversity of structures, microbial origins and roles. Appl. Microbiol. Biotechnol. 86 (5), 1323–1336. 10.1007/s00253-010-2498-2 20336292 PMC2854365

[B2] Altinbag R. C. Ertekin E. Tezel U. (2020). Complete genome sequence of *Pseudomonas* sp. strain BIOMIG1(BAC), which mineralizes benzalkonium chloride disinfectants. Microbiol. Resour. Announc. 9 (20), e00309–e00320. 10.1128/MRA.00309-20 32409540 PMC7225539

[B3] Andersen J. B. (2003). Surface motility in *Pseudomonas* sp. DSS73 is required for efficient biological containment of the root-pathogenic microfungi *Rhizoctonia solani* and *Pythium ultimum* . Microbiology 149 (1), 37–46. 10.1099/mic.0.25859-0 12576578

[B4] Andolfi A. Cimmino A. Cantore P. L. Iacobellis N. S. Evidente A. (2008). Bioactive and structural metabolites of *Pseudomonas* and *Burkholderia* species causal agents of cultivated mushrooms diseases. Perspect. Med. Chem. 2, 1177391X0800200. 10.1177/1177391x0800200004 PMC2746572 19787100

[B5] Arp J. Gotze S. Mukherji R. Mattern D. J. Garcia-Altares M. Klapper M. (2018). Synergistic activity of cosecreted natural products from amoebae-associated bacteria. P Natl. Acad. Sci. U. S. A. 115 (15), 3758–3763. 10.1073/pnas.1721790115 PMC5899472 29592954

[B6] Bahroun A. Jousset A. Mrabet M. Mhamdi R. Mhadhbi H. (2021). Protists modulate Fusarium root rot suppression by beneficial bacteria. Appl. Soil Ecol. 168, 104158. 10.1016/j.apsoil.2021.104158

[B7] Bak F. Bonnichsen L. Jørgensen N. O. Nicolaisen M. H. Nybroe O. (2015). The biosurfactant viscosin transiently stimulates n-hexadecane mineralization by a bacterial consortium. Appl. Microbiol. Biotechnol. 99 (3), 1475–1483. 10.1007/s00253-014-6054-3 25216581 PMC4306737

[B8] Banat I. M. Makkar R. S. Cameotra S. S. (2000). Potential commercial applications of microbial surfactants. Appl. Microbiol. Biotechnol. 53 (5), 495–508. 10.1007/s002530051648 10855707

[B9] Bassarello C. Lazzaroni S. Bifulco G. Lo Cantore P. Iacobellis N. S. Riccio R. (2004). Tolaasins A-E, five new lipodepsipeptides produced by *Pseudomonas tolaasii* . J. Nat. Prod. 67 (5), 811–816. 10.1021/np0303557 15165142

[B10] Batianis C. Kozaeva E. Damalas S. G. Martín-Pascual M. Volke D. C. Nikel P. I. (2020). An expanded CRISPRi toolbox for tunable control of gene expression in *Pseudomonas putida* . Microb. Biotechnol. 13 (2), 368–385. 10.1111/1751-7915.13533 32045111 PMC7017828

[B11] Batoko H. d'Exaerde A. D. Kinet J. M. Bouharmont J. Gage R. A. Maraite H. (1998). Modulation of plant plasma membrane H^+^-ATPase by phytotoxic lipodepsipeptides produced by the plant pathogen *Pseudomonas fuscovaginae* . Biochimica Biophysica Acta-Biomembranes 1372 (2), 216–226. 10.1016/S0005-2736(98)00060-1 9675287

[B12] Bender C. L. Alarcon-Chaidez F. Gross D. C. (1999). *Pseudomonas syringae* phytotoxins: mode of action, regulation, and biosynthesis by peptide and polyketide synthetases. Microbiol. Mol. Biol. Rev. 63 (2), 266–292. 10.1128/MMBR.63.2.266-292.1999 10357851 PMC98966

[B13] Berge O. Monteil C. L. Bartoli C. Chandeysson C. Guilbaud C. Sands D. C. (2014). A user's guide to a data base of the diversity of *Pseudomonas syringae* and its application to classifying strains in this phylogenetic complex. PLoS ONE 9 (9), e105547. 10.1371/journal.pone.0105547 25184292 PMC4153583

[B14] Berry C. L. Brassinga A. K. C. Donald L. J. Fernando W. G. D. Loewen P. C. de Kievit T. R. (2012). Chemical and biological characterization of sclerosin, an antifungal lipopeptide. Can. J. Microbiol. 58 (8), 1027–1034. 10.1139/w2012-079 22838838

[B15] Berry C. L. Nandi M. Manuel J. Brassinga A. K. C. Fernando W. G. D. Loewen P. C. (2014). Characterization of the *Pseudomonas* sp DF41 *quorum* sensing locus and its role in fungal antagonism. Biol. Control 69, 82–89. 10.1016/j.biocontrol.2013.11.005

[B16] Berti A. D. Greve N. J. Christensen Q. H. Thomas M. G. (2007). Identification of a biosynthetic gene cluster and the six associated lipopeptides involved in swarming motility of *Pseudomonas syringae* pv. tomato DC3000. J. Bacteriol. 189 (17), 6312–6323. 10.1128/JB.00725-07 17601782 PMC1951903

[B17] Biessy A. Novinscak A. Blom J. Léger G. Thomashow L. S. Cazorla F. M. (2019). Diversity of phytobeneficial traits revealed by whole-genome analysis of worldwide-isolated phenazine-producing *Pseudomonas* spp. Environ. Microbiol. 21 (1), 437–455. 10.1111/1462-2920.14476 30421490

[B18] Biniarz P. Coutte F. Gancel F. Łukaszewicz M. (2018). High-throughput optimization of medium components and culture conditions for the efficient production of a lipopeptide pseudofactin by Pseudomonas fluorescens BD5. Microb. Cell Factories 17 (1), 121. 10.1186/s12934-018-0968-x PMC6076405 30077177

[B19] Biniarz P. Henkel M. Hausmann R. Łukaszewicz M. (2020). Development of a bioprocess for the production of cyclic lipopeptides pseudofactins with efficient purification from collected foam. Front. Bioeng. Biotechnol. 8, 565619. 10.3389/fbioe.2020.565619 33330412 PMC7719756

[B20] Bonnichsen L. Bygvraa Svenningsen N. Rybtke M. de Bruijn I. Raaijmakers J. M. Tolker-Nielsen T. (2015). Lipopeptide biosurfactant viscosin enhances dispersal of *Pseudomonas fluorescens* SBW25 biofilms. Microbiology 161 (12), 2289–2297. 10.1099/mic.0.000191 26419730 PMC4811653

[B21] Bricout A. Morris C. E. Chandeysson C. Duban M. Boistel C. Chataigne G. (2022). The diversity of lipopeptides in the *Pseudomonas syringae* complex parallels phylogeny and sheds light on structural diversification during evolutionary history. Microbiol. Spectr. 10 (6), e0145622. 10.1128/spectrum.01456-22 36287007 PMC9769872

[B22] Brunetti A. E. Bunk B. Lyra M. L. Fuzo C. A. Marani M. M. Spröer C. (2022). Molecular basis of a bacterial-amphibian symbiosis revealed by comparative genomics, modeling, and functional testing. ISME J. 16 (3), 788–800. 10.1038/s41396-021-01121-7 34601502 PMC8857215

[B23] Bunster L. Fokkema N. J. Schippers B. (1989). Effect of surface-active Pseudomonas spp on leaf wettability. Appl. Environ. Microbiol. 55 (6), 1340–1345. 10.1128/Aem.55.6.1340-1345.1989 16347926 PMC202868

[B24] Burch A. Y. Zeisler V. Yokota K. Schreiber L. Lindow S. E. (2014). The hygroscopic biosurfactant syringafactin produced by *Pseudomonas syringae* enhances fitness on leaf surfaces during fluctuating humidity. Environ. Microbiol. 16 (7), 2086–2098. 10.1111/1462-2920.12437 24571678

[B25] Catara V. (2007). *Pseudomonas corrugata*: plant pathogen and/or biological resource? pathogen profile. Mol. Plant Pathol. 8 (3), 233–244. 10.1111/j.1364-3703.2007.00391.x 20507495

[B26] Cautain B. De Pedro N. Schulz C. Pascual J. Sousa T. D. S. Martin J. (2015). Identification of the lipodepsipeptide MDN-0066, a novel inhibitor of VHL/HIF pathway produced by a new *Pseudomonas* species. PLoS ONE 10 (5), e0125221. 10.1371/journal.pone.0125221 26018559 PMC4445906

[B27] Ceresa C. Fracchia L. Sansotera A. C. De Rienzo M. A. D. Banat I. M. (2023). Harnessing the potential of biosurfactants for biomedical and pharmaceutical applications. Pharmaceutics 15 (8), 2156. 10.3390/pharmaceutics15082156 37631370 PMC10457971

[B28] Cesa-Luna C. Geudens N. Girard L. De Roo V. Maklad H. R. Martins J. C. (2023). Charting the lipopeptidome of nonpathogenic *Pseudomonas* . mSystems 8, e0098822. 10.1128/msystems.00988-22 36719227 PMC9948697

[B29] Chong H. Q. Li Q. X. (2017). Microbial production of rhamnolipids: opportunities, challenges and strategies. Microb. Cell Factories 16, 137. 10.1186/s12934-017-0753-2 PMC5544971 28779757

[B30] Christiansen L. Alanin K. S. Phippen C. B. W. Olsson S. Stougaard P. Hennessy R. C. (2020). Fungal-associated molecules induce key genes involved in the biosynthesis of the antifungal secondary metabolites nunamycin and nunapeptin in the biocontrol strain *Pseudomonas fluorescens* In5. Appl. Environ. Microbiol. 86, 012844. 10.1128/AEM.01284-20 PMC7580543 32826219

[B31] Chwastek G. Surma M. A. Rizk S. Grosser D. Lavrynenko O. Rucińska M. (2020). Principles of membrane adaptation revealed through environmentally induced bacterial lipidome remodeling. Cell Rep. 32 (12), 108165. 10.1016/j.celrep.2020.108165 32966790

[B32] Ciurko D. Chebbi A. Kruszelnicki M. Czapor-Irzabek H. Urbanek A. K. Polowczyk I. (2023). Production and characterization of lipopeptide biosurfactant from a new strain of Pseudomonas Antarctica 28E using crude glycerol as a carbon source. RSC Adv. 13 (34), 24129–24139. 10.1039/D3RA03408A 37577095 PMC10415746

[B33] Cui X. Harling R. Mutch P. Darling D. (2005). Identification of -3-hydroxyoctanoyl-homoserine lactone production in *Pseudomonas fluorescens* 5064, pathogenic to broccoli, and controlling biosurfactant production by *quorum* sensing. Eur. J. Plant Pathology 111 (4), 297–308. 10.1007/s10658-004-4171-z

[B34] D'Aes J. De Maeyer K. Pauwelyn E. Höfte M. (2010). Biosurfactants in plant-*Pseudomonas* interactions and their importance to biocontrol. Environ. Microbiol. Rep. 2 (3), 359–372. 10.1111/j.1758-2229.2009.00104.x 23766108

[B35] D'aes J. Kieu N. P. Léclère V. Tokarski C. Olorunleke F. E. De Maeyer K. (2014). To settle or to move? The interplay between two classes of cyclic lipopeptides in the biocontrol strain *Pseudomonas* CMR12a. Environ. Microbiol. 16 (7), 2282–2300. 10.1111/1462-2920.12462 24673852

[B36] De Almeida D. G. Soares Da Silva R. C. Luna J. M. Rufino R. D. Santos V. A. Banat I. M. (2016). Biosurfactants: promising molecules for petroleum biotechnology advances. Front. Microbiol. 7, 1718. 10.3389/fmicb.2016.01718 27843439 PMC5087163

[B37] de Bruijn I. de Kock M. J. de Waard P. van Beek T. A. Raaijmakers J. M. (2008). Massetolide A biosynthesis in *Pseudomonas fluorescens* . J. Bacteriol. 190 (8), 2777–2789. 10.1128/JB.01563-07 17993540 PMC2293227

[B38] de Bruijn I. de Kock M. J. Yang M. de Waard P. van Beek T. A. Raaijmakers J. M. (2007). Genome-based discovery, structure prediction and functional analysis of cyclic lipopeptide antibiotics in *Pseudomonas* species. Mol. Microbiol. 63 (2), 417–428. 10.1111/j.1365-2958.2006.05525.x 17241198

[B39] de Bruijn I. Raaijmakers J. M. (2009). Diversity and functional analysis of LuxR-type transcriptional regulators of cyclic lipopeptide biosynthesis in *Pseudomonas fluorescens* . Appl. Environ. Microbiol. 75 (14), 4753–4761. 10.1128/AEM.00575-09 19447950 PMC2708414

[B40] de Cássia F. S. S. R. Almeida D. G. Rufino R. D. Luna J. M. Santos V. A. Sarubbo L. A. (2014). Applications of biosurfactants in the petroleum industry and the remediation of oil spills. Int. J. Mol. Sci. 15 (7), 12523–12542. 10.3390/ijms150712523 25029542 PMC4139858

[B41] De Roo V. Verleysen Y. Kovács B. Vleeschouwer M. D. Muangkaew P. Girard L. (2022). An nuclear magnetic resonance fingerprint matching approach for the identification and structural Re-evaluation of *Pseudomonas* lipopeptides. Microbiol. Spectr. 10 (4), e0126122. 10.1128/spectrum.01261-22 35876524 PMC9431178

[B42] De Vleeschouwer M. Martins J. Madder A. (2016). First total synthesis of WLIP: on the importance of correct protecting group choice. J. Peptide Sci. 22 (3), 149–155. 10.1002/psc.2852 26856688

[B43] De Vleeschouwer M. Sinnaeve D. den Begin J. Coenye T. Martins J. Madder A. (2014). Rapid total synthesis of cyclic lipodepsipeptides as a premise to investigate their self-assembly and biological activity. Chem. - A Eur. J. 20 (25), 7766–7775. 10.1002/chem.201402066 24817328

[B44] Dubern J. F. Coppoolse E. R. Stiekema W. J. Bloemberg G. V. (2008). Genetic and functional characterization of the gene cluster directing the biosynthesis of putisolvin I and II in *Pseudomonas putida* strain PCL1445. Microbiology 154, 2070–2083. 10.1099/mic.0.2008/016444-0 18599835

[B45] Dubern J. F. Lagendijk E. L. Lugtenberg B. J. J. Bloemberg G. V. (2005). The heat shock genes *dnaK, dnaJ,* and *grpE* are involved in regulation of putisolvin biosynthesis in *Pseudomonas putida* PCL1445. J. Bacteriol. 187 (17), 5967–5976. 10.1128/Jb.187.17.5967-5976.2005 16109938 PMC1196155

[B46] Dubern J. F. Lugtenberg B. J. Bloemberg G. V. (2006). The *ppuI-rsaL-ppuR quorum*-sensing system regulates biofilm formation of *Pseudomonas putida* PCL1445 by controlling biosynthesis of the cyclic lipopeptides putisolvins I and II. J. Bacteriol. 188 (8), 2898–2906. 10.1128/JB.188.8.2898-2906.2006 16585751 PMC1447005

[B47] Dufour S. Deleu M. Nott K. Wathelet B. Thonart P. Paquot M. (2005). Hemolytic activity of new linear surfactin analogs in relation to their physico-chemical properties. Biochimica Biophysica Acta (BBA) - General Subj. 1726 (1), 87–95. 10.1016/j.bbagen.2005.06.015 16026933

[B48] Eeman M. Berquand A. Dufrêne Y. F. Paquot M. Dufour S. Deleu M. (2006). Penetration of surfactin into phospholipid monolayers: nanoscale interfacial organization. Langmuir 22 (26), 11337–11345. 10.1021/la061969p 17154623

[B49] Ferrarini E. Roo V. D. Geudens N. Martins J. C. Höfte M. (2022a). Altering *in vivo* membrane sterol composition affects the activity of the cyclic lipopeptides tolaasin and sessilin against *Pythium* . Biochimica Biophysica Acta - Biomembr. 1864, 184008. 10.1016/j.bbamem.2022.184008 35868404

[B50] Ferrarini E. Spacapan M. Lam V. B. McCann A. Cesa-Luna C. Marahatta B. (2022b). Versatile role of *Pseudomonas fuscovaginae* cyclic lipopeptides in plant and microbial interactions. Front. Plant Sci. 13, 1008980. 10.3389/fpls.2022.1008980 36426159 PMC9679282

[B51] Fitzpatrick A. W. P. Llabres S. Neuberger A. Blaza J. N. Bai X. C. Okada U. (2017). Structure of the MacAB-TolC ABC-type tripartite multidrug efflux pump. Nat. Microbiol. 2, 17070. 10.1038/nmicrobiol.2017.70 28504659 PMC5447821

[B52] Flury P. Vesga P. Péchy-Tarr M. Aellen N. Dennert F. Hofer N. (2017). Antimicrobial and insecticidal: cyclic lipopeptides and hydrogen cyanide produced by plant-beneficial *Pseudomonas strains* CHA0, CMR12a, and PCL1391 contribute to insect killing. Front. Microbiol. 8 (100), 100. 10.3389/fmicb.2017.00100 28217113 PMC5289993

[B53] Fogliano V. Ballio A. Gallo M. Woo S. Scala F. Lorito M. (2002). *Pseudomonas* lipodepsipeptides and fungal cell wall-degrading enzymes act synergistically in biological control. Mol. Plant-Microbe Interact. 15 (4), 323–333. 10.1094/Mpmi.2002.15.4.323 12026170

[B54] Gao Z. Karlsson I. Geisen S. Kowalchuk G. Jousset A. (2019). Protists: puppet masters of the rhizosphere microbiome. Trends Plant Sci. 24 (2), 165–176. 10.1016/j.tplants.2018.10.011 30446306

[B55] Geudens N. Martins J. C. (2018). Cyclic lipodepsipeptides from *Pseudomonas* spp – biological Swiss-army knives. Front. Microbiol. 9, 1867. 10.3389/fmicb.2018.01867 30158910 PMC6104475

[B56] Girard L. Geudens N. Pauwels B. Höfte M. Martins J. C. De Mot R. (2022). Transporter gene-mediated typing for detection and genome mining of lipopeptide-producing *Pseudomonas* . Appl. Environ. Microbiol. 88 (2), e0186921. 10.1128/AEM.01869-21 34731056 PMC8788793

[B57] Girard L. Höfte M. De Mot R. (2020). Lipopeptide families at the interface between pathogenic and beneficial *Pseudomonas*-plant interactions. Crit. Rev. Microbiol. 46 (4), 397–419. 10.1080/1040841X.2020.1794790 32885723

[B58] Girard L. Lood C. Höfte M. Vandamme P. Rokni-Zadeh H. Van Noort V. (2021). The ever-expanding *Pseudomonas* genus: description of 43 new species and partition of the *Pseudomonas putida* group. Microorganisms 9, 1766. 10.3390/microorganisms9081766 34442845 PMC8401041

[B59] Gislason A. S. de Kievit T. R. (2020). Friend or foe? Exploring the fine line between *Pseudomonas brassicacearum* and phytopathogens. J. Med. Microbiol. 69 (3), 347–360. 10.1099/jmm.0.001145 31976855

[B60] Götze S. Stallforth P. (2020). Structure, properties, and biological functions of nonribosomal lipopeptides from pseudomonads. Nat. Product. Rep. 37 (1), 29–54. 10.1039/c9np00022d 31436775

[B61] Götze S. Vij R. Burow K. Thome N. Urbat L. Schlosser N. (2023). Ecological niche-inspired genome mining leads to the discovery of crop-protecting nonribosomal lipopeptides featuring a transient amino acid building block. J. Am. Chem. Soc. 145 (4), 2342–2353. 10.1021/jacs.2c11107 36669196 PMC9897216

[B62] Gram L. (2015). Silent clusters - speak up. Microb. Biotechnol. 8 (1), 13–14. 10.1111/1751-7915.12181 25545918 PMC4321359

[B63] Grewal S. I. S. Han B. Johnstone K. (1995). Identification and characterization of a locus which regulates multiple functions in *Pseudomonas tolaasii*, the cause of Brown blotch disease of *Agaricus bisporus* . J. Bacteriol. 177 (16), 4658–4668. 10.1128/jb.177.16.4658-4668.1995 7642492 PMC177230

[B64] Grgurina I. Gross D. C. Iacobellis N. S. Lavermicocca P. Takemoto J. Y. Benincasa M. (1996). Phytotoxin production by *Pseudomonas syringae* pv. syringae: syringopeptin production by syr mutants defective in biosynthesis or secretion of syringomycin. FEMS Microbiol. Lett. 138 (1), 35–39. 10.1111/j.1574-6968.1996.tb08131.x

[B65] Gross D. C. (1985). Regulation of syringomycin synthesis in *Pseudomonas syringae* pv. *syringae* and defined conditions for its production. J. Appl. Bacteriol. 58 (2), 167–174. 10.1111/j.1365-2672.1985.tb01444.x 3980301

[B66] Gross D. C. DeVay J. E. (1977). Production and purification of syringomycin, a phytotoxin produced by *Pseudomonas syringae* . Physiol. Plant Pathol. 11 (1), 13–28. 10.1016/0048-4059(77)90083-2

[B67] Gross H. Stockwell V. O. Henkels M. D. Nowak-Thompson B. Loper J. E. Gerwick W. H. (2007). The genomisotopic approach: a systematic method to isolate products of orphan biosynthetic gene clusters. Chem. Biol. 14 (1), 53–63. 10.1016/j.chembiol.2006.11.007 17254952

[B68] Gu Y.-L. Li J.-Z. Li Y. Cong S. Wang J. Ma Y.-N. (2023). *Pseudomonas* cyclic lipopeptide medpeptin: biosynthesis and modulation of plant immunity. Engineering 28, 153–165. 10.1016/j.eng.2023.05.016

[B69] Guez J. S. Vassaux A. Larroche C. Jacques P. Coutte F. (2021). New continuous process for the production of lipopeptide biosurfactants in foam overflowing bioreactor. Front. Bioeng. Biotechnol. 9, 678469. 10.3389/fbioe.2021.678469 34124025 PMC8194703

[B70] Guo S. Xiong W. Hang X. Gao Z. Jiao Z. Liu H. (2021). Protists as main indicators and determinants of plant performance. Microbiome 9 (1), 64. 10.1186/s40168-021-01025-w 33743825 PMC7981826

[B71] Gutiérrez-Chávez C. Benaud N. Ferrari B. C. (2021). The ecological roles of microbial lipopeptides: where are we going? Comput. Struct. Biotechnol. J. 19, 1400–1413. 10.1016/j.csbj.2021.02.017 33777336 PMC7960500

[B72] Haas D. Défago G. (2005). Biological control of soil-borne pathogens by fluorescent pseudomonads. Nat. Rev. Microbiol. 3 (4), 307–319. 10.1038/nrmicro1129 15759041

[B73] Han B. Pain A. Johnstone K. (1997). Spontaneous duplication of a 661 bp element within a two-component sensor regulator gene causes phenotypic switching in colonies of *Pseudomonas tolaasii*, cause of brown blotch disease of mushrooms. Mol. Microbiol. 25 (2), 211–218. 10.1046/j.1365-2958.1997.4411811.x 9282733

[B74] Hansen M. L. Dénes Z. Jarmusch S. A. Wibowo M. Lozano-Andrade C. N. Kovács Á. T. (2023). Resistance towards and biotransformation of Pseudomonas-produced secondary metabolites during community invasion. BioRxiv. 10.1101/2023.06.20.545698 PMC11203913 38874164

[B75] Hawxhurst C. J. Micciulla J. L. Bridges C. M. Shor M. Gage D. J. Shor L. M. (2023). Soil protists can actively redistribute beneficial bacteria along *Medicago truncatula roots* . Appl. Environ. Microbiol. 89 (3), e0181922. 10.1128/aem.01819-22 36877040 PMC10057870

[B76] Heeb S. Haas D. (2001). Regulatory roles of the GacS/GacA two-component system in plant-associated and other Gram-negative bacteria. Mol. Plant-Microbe Interact. 14 (12), 1351–1363. 10.1094/Mpmi.2001.14.12.1351 11768529

[B77] Hennessy R. C. Phippen C. B. W. Nielsen K. F. Olsson S. Stougaard P. (2017a). Biosynthesis of the antimicrobial cyclic lipopeptides nunamycin and nunapeptin by *Pseudomonas fluorescens* strain In5 is regulated by the LuxR-type transcriptional regulator NunF. Microbiologyopen 6 (6), e00516. 10.1002/mbo3.516 28782279 PMC5727362

[B78] Hennessy R. C. Stougaard P. Olsson S. (2017b). A microplate reader-based system for visualizing transcriptional activity during *in vivo* microbial interactions in space and time. Sci. Rep. 7 (1), 281. 10.1038/s41598-017-00296-4 28325928 PMC5412646

[B79] Hermenau R. Kugel S. Komor A. J. Hertweck C. (2020). Helper bacteria halt and disarm mushroom pathogens by linearizing structurally diverse cyclolipopeptides. P Natl. Acad. Sci. U. S. A. 117 (38), 23802–23806. 10.1073/pnas.2006109117 PMC7519232 32868430

[B80] Hildebrand P. D. Braun P. G. McRae K. B. Lu X. (1998). Role of the biosurfactant viscosin in broccoli head rot caused by a pectolytic strain of*Pseudomonas fluorescens* . Can. J. Plant Pathology 20 (3), 296–303. 10.1080/07060669809500396

[B81] Hockett K. L. Burch A. Y. Lindow S. E. (2013). Thermo-regulation of genes mediating motility and plant interactions in *Pseudomonas syringae* . Plos One 8 (3), e59850. 10.1371/journal.pone.0059850 23527276 PMC3602303

[B82] Höfte M. (2021). “The use of Pseudomonas spp. as bacterial biocontrol agents to control plant disease,” in Microbial bioprotectants for plant disease management. Editors Köhl, J. Burleigh Dodds W. R. (Cambridge: Burleigh Dodds Science Publishing), 301–374.

[B83] Hrabak E. M. Willis D. K. (1992). The *lemA* gene required for pathogenicity of *Pseudomonas syringae* pv. syringae on bean is a member of a family of two-component regulators. J. Bacteriol. 174 (9), 3011–3020. 10.1128/jb.174.9.3011-3020.1992 1314807 PMC205956

[B84] Hrabak E. M. Willis D. K. (1993). Involvement of the *IemA* gene in production of syringomycin and protease by *Pseudomonas syringae* pv *syringae* . Mol. Plant-Microbe Interact. 6 (3), 368–375. 10.1094/Mpmi-6-368

[B85] Huang C. J. Pauwelyn E. Ongena M. Debois D. Leclère V. Jacques P. (2015). Characterization of cichopeptins, new phytotoxic cyclic lipodepsipeptides produced by *Pseudomonas cichorii* SF1-54 and their role in bacterial midrib rot disease of lettuce. Mol. Plant-Microbe Interact. 28 (9), 1009–1022. 10.1094/MPMI-03-15-0061-R 25961750

[B86] Humair B. Wackwitz B. Haas D. (2010). GacA-controlled activation of promoters for small RNA genes in *Pseudomonas fluorescens* . Appl. Environ. Microbiol. 76 (5), 1497–1506. 10.1128/Aem.02014-09 20048056 PMC2832403

[B87] Hutchison M. L. Johnstone K. (1993). Evidence for the involvement of the surface-active properties of the extracellular toxin tolaasin in the manifestation of Brown blotch disease symptoms by *Pseudomonas tolaasii* on *Agaricus bisporus* . Physiological Mol. Plant Pathology 42 (5), 373–384. 10.1016/S0885-5765(05)80013-X

[B88] Jahanshah G. Yan Q. Gerhardt H. Pataj Z. Lämmerhofer M. Pianet I. (2019). Discovery of the cyclic lipopeptide gacamide A by genome mining and repair of the defective GacA regulator in *Pseudomonas fluorescens* Pf0-1. J. Nat. Prod. 82 (2), 301–308. 10.1021/acs.jnatprod.8b00747 30666877

[B89] Janek T. Krasowska A. Radwańska A. Łukaszewicz M. (2013). Lipopeptide biosurfactant pseudofactin II induced apoptosis of melanoma A 375 cells by specific interaction with the plasma membrane. PLoS ONE 8 (3), e57991–e57999. 10.1371/journal.pone.0057991 23483962 PMC3590301

[B90] Janek T. Łukaszewicz M. Rezanka T. Krasowska A. (2010). Isolation and characterization of two new lipopeptide biosurfactants produced by Pseudomonas fluorescens BD5 isolated from water from the Arctic Archipelago of Svalbard. Bioresour. Technol. 101 (15), 6118–6123. 10.1016/j.biortech.2010.02.109 20303744

[B91] Jang J. Y. Yang S. Y. Kim Y. C. Lee C. W. Park M. S. Kim J. C. (2013). Identification of orfamide A as an insecticidal metabolite produced by Pseudomonas protegens F6. J. Agric. Food Chem. 61 (28), 6786–6791. 10.1021/jf401218w 23763636

[B92] Ji X. J. Nielsen A. L. Heinis C. (2023). Cyclic peptides for drug development. Angew. Chem. Int. Ed. 63, e202308251. 10.1002/anie.202308251 37870189

[B93] Johnston C. W. Skinnider M. A. Wyatt M. A. Li X. Ranieri M. R. M. Yang L. (2015). An automated Genomes-to-Natural Products platform (GNP) for the discovery of modular natural products. Nat. Commun. 6, 8421. 10.1038/ncomms9421 26412281 PMC4598715

[B94] Kang H. J. Gross D. C. (2005). Characterization of a resistance-nodulation-cell division transporter system associated with the syr-syp genomic island of *Pseudomonas syringae* pv. *syringae* . Appl. Environ. Microbiol. 71 (9), 5056–5065. 10.1128/Aem.71.9.5056-5065.2005 16151087 PMC1214623

[B95] Kitten T. Kinscherf T. G. McEvoy J. L. Willis D. K. (1998). A newly identified regulator is required for virulence and toxin production in *Pseudomonas syringae* . Mol. Microbiol. 28 (5), 917–929. 10.1046/j.1365-2958.1998.00842.x 9663679

[B96] Koch B. Nielsen T. H. Sorensen D. Andersen J. B. Christophersen C. Molin S. (2002). Lipopeptide production in *Pseudomonas* sp. strain DSS73 is regulated by components of sugar beet seed exudate via the Gac two-component regulatory system. Appl. Environ. Microbiol. 68 (9), 4509–4516. 10.1128/AEM.68.9.4509-4516.2002 12200307 PMC124083

[B97] Korbut R. Skjolding L. M. Mathiessen H. Jaafar R. Li X. Jørgensen L. V. G. (2022). Toxicity of the antiparasitic lipopeptide biosurfactant SPH6 to green algae, cyanobacteria, crustaceans and zebrafish. Aquat. Toxicol. 243, 106072. 10.1016/j.aquatox.2021.106072 35032912

[B98] Kruijt M. Tran H. Raaijmakers J. M. (2009). Functional, genetic and chemical characterization of biosurfactants produced by plant growth-promoting*Pseudomonas putida*267. J. Appl. Microbiol. 107 (2), 546–556. 10.1111/j.1365-2672.2009.04244.x 19302489

[B99] Kuiper I. Lagendijk E. L. Pickford R. Derrick J. P. Lamers G. E. M. Thomas-Oates J. E. (2004). Characterization of two *Pseudomonas putida* lipopeptide biosurfactants, putisolvin I and II, which inhibit biofilm formation and break down existing biofilms. Mol. Microbiol. 51 (1), 97–113. 10.1046/j.1365-2958.2003.03751.x 14651614

[B100] Lavermicocca P. Iacobellis N. S. Simmaco M. Graniti A. (1997). Biological properties and spectrum of activity of *Pseudomonas syringae* pv *syringae* toxins. Physiological Mol. Plant Pathology 50 (2), 129–140. 10.1006/pmpp.1996.0078

[B101] Leonov P. S. Flores-Alsina X. Gernaey K. V. Sternberg C. (2021). Microbial biofilms in biorefinery - towards a sustainable production of low-value bulk chemicals and fuels. Biotechnol. Adv. 50, 107766. 10.1016/j.biotechadv.2021.107766 33965529

[B102] Li W. Rokni-Zadeh H. De Vleeschouwer M. Ghequire M. G. K. K. Sinnaeve D. Xie G.-L. L. (2013). The antimicrobial compound xantholysin defines a new group of *Pseudomonas* cyclic lipopeptides. PLoS ONE 8 (5), e62946. 10.1371/journal.pone.0062946 23690965 PMC3656897

[B103] Licciardello G. Bertani I. Steindler L. Bella P. Venturi V. Catara V. (2009). The transcriptional activator *rfiA* is *quorum*-sensing regulated by cotranscription with the *luxI* homolog *pcoI* and is essential for plant virulence in *Pseudomonas corrugata* . Mol. Plant-Microbe Interact. 22 (12), 1514–1522. 10.1094/MPMI-22-12-1514 19888817

[B104] Liu J. F. Mbadinga S. M. Yang S. Z. Gu J. D. Mu B. Z. (2015). Chemical structure, property and potential applications of biosurfactants produced by *Bacillus subtilis* in petroleum recovery and spill mitigation. Int. J. Mol. Sci. 16 (3), 4814–4837. 10.3390/ijms16034814 25741767 PMC4394451

[B105] Lo Cantore P. Lazzaroni S. Coraiola M. Dalla Serra M. Cafarchia C. Evidente A. (2006). Biological characterization of white line-inducing principle (WLIP) produced by *Pseudomonas reactans* NCPPB1311. Mol. Plant Microbe Interact. 19 (10), 1113–1120. 10.1094/mpmi-19-1113 17022175

[B106] Lu S. E. Scholz-Schroeder B. K. Gross D. C. (2002). Characterization of the *salA, syrF*, and *syrG* regulatory genes located at the right border of the syringomycin gene cluster of *Pseudomonas syringae* pv. *syringae* . Mol. Plant-Microbe Interact. 15 (1), 43–53. 10.1094/MPMI.2002.15.1.43 11843302

[B107] Lu S. E. Wang N. Wang J. Chen Z. J. Gross D. C. (2005). Oligonucleotide microarray analysis of the *salA* regulon controlling phytotoxin production by *Pseudomonas syringae* pv. *syringae* . Mol. Plant-Microbe Interact. 18 (4), 324–333. 10.1094/MPMI-18-0324 15828684

[B108] Ma Z. Geudens N. Kieu N. P. Sinnaeve D. Ongena M. Martins J. C. (2016a). Biosynthesis, chemical structure, and structure-activity relationship of orfamide lipopeptides produced by *Pseudomonas protegens* and related species. Front. Microbiol. 7, 382. 10.3389/fmicb.2016.00382 27065956 PMC4811929

[B109] Ma Z. Hua G. K. H. Ongena M. Höfte M. (2016b). Role of phenazines and cyclic lipopeptides produced by *Pseudomonas* sp. CMR12a in induced systemic resistance on rice and bean. Environ. Microbiol. Rep. 8 (5), 896–904. 10.1111/1758-2229.12454 27557735

[B110] Ma Z. Ongena M. Höfte M. (2017). The cyclic lipopeptide orfamide induces systemic resistance in rice to *Cochliobolus miyabeanus* but not to *Magnaporthe oryzae* . Plant Cell Rep. 36 (11), 1731–1746. 10.1007/s00299-017-2187-z 28801742

[B111] Martin C. Ibáñez R. Nothias L.-F. Boya P C. A. Reinert L. K. Rollins-Smith L. A. (2019). Viscosin-like lipopeptides from frog skin bacteria inhibit *Aspergillus fumigatus* and *Batrachochytrium dendrobatidis* detected by imaging mass spectrometry and molecular networking. Sci. Rep. 9 (1), 3019. 10.1038/s41598-019-39583-7 30816229 PMC6395710

[B112] Mavrodi D. V. Peever T. L. Mavrodi O. V. Parejko J. A. Raaijmakers J. M. Lemanceau P. (2010). Diversity and evolution of the phenazine biosynthesis pathway. Appl. Environ. Microbiol. 76 (3), 866–879. 10.1128/AEM.02009-09 20008172 PMC2813009

[B113] Mazzola M. de Bruijn I. Cohen M. F. Raaijmakers J. M. (2009). Protozoan-induced regulation of cyclic lipopeptide biosynthesis is an effective predation defense mechanism for *Pseudomonas fluorescens* . Appl. Environ. Microbiol. 75 (21), 6804–6811. 10.1128/AEM.01272-09 19717630 PMC2772446

[B114] Meleshko D. Mohimani H. Tracanna V. Hajirasouliha I. Medema M. H. Korobeynikov A. (2019). BiosyntheticSPAdes: reconstructing biosynthetic gene clusters from assembly graphs. Genome Res. 29 (8), 1352–1362. 10.1101/gr.243477.118 31160374 PMC6673720

[B115] Melnyk R. A. Hossain S. S. Haney C. H. (2019). Convergent gain and loss of genomic islands drive lifestyle changes in plant-associated *Pseudomonas* . ISME J. 13 (6), 1575–1588. 10.1038/s41396-019-0372-5 30787396 PMC6776051

[B116] Mendes R. Kruijt M. De Bruijn I. Dekkers E. Van Der Voort M. Schneider J. H. M. (2011). Deciphering the rhizosphere microbiome for disease-suppressive bacteria. Science 332 (6033), 1097–1100. 10.1126/science.1203980 21551032

[B117] Michelsen C. F. Jensen H. Venditto V. J. Hennessy R. C. Stougaard P. (2015a). Bioactivities by a crude extract from the Greenlandic *Pseudomonas* sp. In5 involves the nonribosomal peptides, nunamycin and nunapeptin. PeerJ 3, e1476. 10.7717/peerj.1476 26734508 PMC4699791

[B118] Michelsen C. F. Stougaard P. (2011). A novel antifungal *Pseudomonas fluorescens* isolated from potato soils in Greenland. Curr. Microbiol. 62 (4), 1185–1192. 10.1007/s00284-010-9846-4 21165740

[B119] Michelsen C. F. Watrous J. Glaring M. A. Kersten R. Koyama N. Dorrestein P. C. (2015b). Nonribosomal peptides, key biocontrol components for *Pseudomonas fluorescens* In5, isolated from a Greenlandic suppressive soil. mBio 6 (2), e00079–e00015. 10.1128/mBio.00079-15 25784695 PMC4453515

[B120] Monfil V. O. Casas-Flores S. (2014). “Chapter 32 - molecular mechanisms of biocontrol in Trichoderma spp. and their applications in agriculture,” in Biotechnology and biology of Trichoderma. Editors Gupta V. K. Schmoll M. Herrera-Estrella A. Upadhyay R. S. Druzhinina I. Tuohy M. G. (Amsterdam: Elsevier), 429–453.

[B121] Muangkaew P. De Roo V. Zhou L. Girard L. Cesa-Luna C. Hofte M. (2023). Stereomeric lipopeptides from a single non-ribosomal peptide synthetase as an additional source of structural and functional diversification in *Pseudomonas* lipopeptide biosynthesis. Int. J. Mol. Sci. 24 (18), 14302. 10.3390/ijms241814302 37762605 PMC10531924

[B122] Murata H. Tsukamoto T. Shirata A. (1998). *rtpA*, a gene encoding a bacterial two-component sensor kinase, determines pathogenic traits of *Pseudomonas tolaasii*, the causal agent of brown blotch disease of a cultivated mushroom. Pleurotus Ostreatus. Mycoscience 39, 261–271. 10.1007/bf02464007

[B123] Nielsen T. H. Christophersen C. Anthoni U. Sorensen J. (1999). Viscosinamide, a new cyclic depsipeptide with surfactant and antifungal properties produced by *Pseudomonas fluorescens* DR54. J. Appl. Microbiol. 87 (1), 80–90. 10.1046/j.1365-2672.1999.00798.x 10432590

[B124] Nielsen T. H. Sørensen D. Tobiasen C. Andersen J. B. Christophersen C. Givskov M. (2002). Antibiotic and biosurfactant properties of cyclic lipopeptides produced by fluorescent *Pseudomonas* spp. from the sugar beet rhizosphere. Appl. Environ. Microbiol. 68 (7), 3416–3423. 10.1128/aem.68.7.3416-3423.2002 12089023 PMC126818

[B125] Nielsen T. H. Sorensen J. (2003). Production of cyclic lipopeptides by *Pseudomonas fluorescens* strains in bulk soil and in the sugar beet rhizosphere. Appl. Environ. Microbiol. 69 (2), 861–868. 10.1128/Aem.69.2.861-868.2003 12571005 PMC143599

[B126] Nielsen T. H. Thrane C. Christophersen C. Anthoni U. Sorensen J. (2000). Structure, production characteristics and fungal antagonism of tensin - a new antifungal cyclic lipopeptide from *Pseudomonas fluorescens* strain 96.578. J. Appl. Microbiol. 89 (6), 992–1001. 10.1046/j.1365-2672.2000.01201.x 11123472

[B127] Nikel P. I. Martínez-García E. de Lorenzo V. (2014). Biotechnological domestication of pseudomonads using synthetic biology. Nat. Rev. Microbiol. 12 (5), 368–379. 10.1038/nrmicro3253 24736795

[B128] Ntana F. Hennessy R. C. Zervas A. Stougaard P. (2023). *Pseudomonas nunensis* sp. nov. isolated from a suppressive potato field in Greenland. Int. J. Syst. Evol. Microbiol. 73 (2), 005700. 10.1099/ijsem.0.005700 36749687

[B129] Nybroe O. Sorensen J. (2004). “Production of cyclic lipopeptides by fluorescent pseudomonads,” in Pseudomonas, biosynthesis of macromolecules and molecular metabolism. Editor Ramos J.-L. (Kluwer Academic/Plenum), 147–172.

[B130] Olorunleke F. E. Hua G. K. H. Kieu N. P. Ma Z. Höfte M. (2015). Interplay between orfamides, sessilins and phenazines in the control of *Rhizoctonia* diseases by *Pseudomonas* sp. CMR12a. Environ. Microbiol. Rep. 7 (5), 774–781. 10.1111/1758-2229.12310 26085277

[B131] Olorunleke F. E. Kieu N. P. De Waele E. Timmerman M. Ongena M. Höfte M. (2017). Coregulation of the cyclic lipopeptides orfamide and sessilin in the biocontrol strain *Pseudomonas* sp CMR12a. Microbiologyopen 6 (5), e00499. 10.1002/mbo3.499 28621084 PMC5635164

[B132] Omoboye O. O. (2019). Cyclic lipopeptide diversity and biocontrol versatility of Pseudomonas species associated with the cocoyam rhizospere. PhD Thesis. Ghent: Ghent University.

[B133] Omoboye O. O. Geudens N. Duban M. Chevalier M. Flahaut C. Martins J. C. (2019a). *Pseudomonas* sp. COW3 produces new bananamide-type cyclic lipopeptides with antimicrobial activity against *Pythium myriotylum* and *Pyricularia oryzae* . Molecules 24, 4170. 10.3390/molecules24224170 31744250 PMC6891508

[B134] Omoboye O. O. Oni F. E. Batool H. Yimer H. Z. De Mot R. Höfte M. (2019b). *Pseudomonas* cyclic lipopeptides suppress the rice blast fungus *Magnaporthe oryzae* by induced resistance and direct antagonism. Front. Plant Sci. 10, 901. 10.3389/fpls.2019.00901 31354771 PMC6636606

[B135] Oni F. E. Esmaeel Q. Onyeka J. T. Adeleke R. Jacquard C. Clement C. (2022). Pseudomonas lipopeptide-mediated biocontrol: chemotaxonomy and biological activity. Molecules 27 (2), 372. 10.3390/molecules27020372 35056688 PMC8777863

[B136] Oni F. E. Geudens N. Adiobo A. Omoboye O. O. Enow E. A. Onyeka J. T. (2020a). Biosynthesis and antimicrobial activity of pseudodesmin and viscosinamide cyclic lipopeptides produced by pseudomonads associated with the cocoyam rhizosphere. Microorganisms 8 (7), 1079. 10.3390/microorganisms8071079 32698413 PMC7409209

[B137] Oni F. E. Geudens N. Omoboye O. O. Bertier L. Hua H. G. K. Adiobo A. (2019a). Fluorescent *Pseudomonas* and cyclic lipopeptide diversity in the rhizosphere of cocoyam (*Xanthosoma sagittifolium*). Environ. Microbiol. 21 (3), 1019–1034. 10.1111/1462-2920.14520 30623562

[B138] Oni F. E. Geudens N. Onyeka J. T. Olorunleke O. F. Salami A. E. Omoboye O. O. (2020b). Cyclic lipopeptide-producing *Pseudomonas koreensis* group strains dominate the cocoyam rhizosphere of a *Pythium* root rot suppressive soil contrasting with *P. putida* prominence in conducive soils. Environ. Microbiol. 22 (12), 5137–5155. 10.1111/1462-2920.15127 32524747

[B139] Oni F. E. Olorunleke O. F. Höfte M. (2019b). Phenazines and cyclic lipopeptides produced by *Pseudomonas* sp. CMR12a are involved in the biological control of *Pythium myriotylum* on cocoyam (*Xanthosoma sagittifolium*). Biol. control 129, 109–114. 10.1016/j.biocontrol.2018.10.005

[B140] Osdaghi E. Martins S. J. Ramos-Sepulveda L. Vieira F. R. Pecchia J. A. Beyer D. M. (2019). 100 Years since tolaas: bacterial blotch of mushrooms in the 21st century. Plant Dis. 103 (11), 2714–2732. 10.1094/Pdis-03-19-0589-Fe 31560599

[B141] Pacheco-Moreno A. Stefanato F. L. Ford J. J. Trippel C. Uszkoreit S. Ferrafiat L. (2021). Pan-genome analysis identifies intersecting roles for Pseudomonas specialized metabolites in potato pathogen inhibition. eLife 10, e71900. 10.7554/eLife.71900 34792466 PMC8719888

[B142] Pascual J. García-López M. Carmona C. Sousa T. d.S. de Pedro N. Cautain B. (2014). *Pseudomonas soli* sp. nov., a novel producer of xantholysin congeners. Syst. Appl. Microbiol. 37 (6), 412–416. 10.1016/j.syapm.2014.07.003 25097020

[B143] Pauwelyn E. Huang C. J. Ongena M. Leclère V. Jacques P. Bleyaert P. (2013). New linear lipopeptides produced by *Pseudomonas cichorii* SF1-54 are involved in virulence, swarming motility, and biofilm formation. Mol. Plant-Microbe Interact. 26 (5), 585–598. 10.1094/MPMI-11-12-0258-R 23405865

[B144] Peschel A. Sahl H. G. (2006). The co-evolution of host cationic antimicrobial peptides and microbial resistance. Nat. Rev. Microbiol. 4 (7), 529–536. 10.1038/nrmicro1441 16778838

[B145] Pflanze S. Mukherji R. Ibrahim A. Gunther M. Gotze S. Chowdhury S. (2023). Nonribosomal peptides protect *Pseudomonas nunensis* 4A2e from amoebal and nematodal predation. Chem. Sci. 14 (41), 11573–11581. 10.1039/d3sc03335j 37886094 PMC10599466

[B146] Pršić J. Ongena M. (2020). Elicitors of plant immunity triggered by beneficial bacteria. Front. Plant Sci. 11, 594530. 10.3389/fpls.2020.594530 33304371 PMC7693457

[B147] Quigley N. B. Gross D. C. (1994). Syringomycin production among strains of *Pseudomonas syringae* pv *syringae* - conservation of the *syrB* and *syrD* genes and activation of phytotoxin production by plant signal molecules. Mol. Plant-Microbe Interact. 7 (1), 78–90. 10.1094/Mpmi-7-0078 7909458

[B148] Quigley N. B. Mo Y. Y. Gross D. C. (1993). SyrD is required for syringomycin production by *Pseudomonas syringae* pathovar *syringae* and is related to a family of ATP-binding secretion proteins. Mol. Microbiol. 9 (4), 787–801. 10.1111/j.1365-2958.1993.tb01738.x 8231810

[B149] Raaijmakers J. M. de Bruijn I. de Kock M. J. D. (2006). Cyclic lipopeptide production by plant-associated *Pseudomonas* spp.: diversity, activity, biosynthesis, and regulation. Mol. Plant-Microbe Interact. 19 (7), 699–710. 10.1094/mpmi-19-0699 16838783

[B150] Raaijmakers J. M. de Bruijn I. Nybroe O. Ongena M. (2010). Natural functions of lipopeptides from *Bacillus* and *Pseudomonas*: more than surfactants and antibiotics. FEMS Microbiol. Rev. 34 (6), 1037–1062. 10.1111/j.1574-6976.2010.00221.x 20412310

[B151] Rainey P. B. Brodey C. L. Johnstone K. (1991). Biological properties and spectrum of activity of tolaasin, a lipodepsipeptide toxin produced by the mushroom pathogen Pseudomonas tolaasii. Physiological Mol. Plant Pathology 39 (1), 57–70. 10.1016/0885-5765(91)90031-C

[B152] Raj A. Kumar A. Dames J. F. (2021). Tapping the role of microbial biosurfactants in pesticide remediation: an eco-friendly approach for environmental sustainability. Front. Microbiol. 12, 791723. 10.3389/fmicb.2021.791723 35003022 PMC8733403

[B153] Reddy C. A. Saravanan R. S. (2013). Polymicrobial multi-functional approach for enhancement of crop productivity. Adv. Appl. Microbiol. 82, 53–113. 10.1016/b978-0-12-407679-2.00003-x 23415153

[B154] Reen F. J. Romano S. Dobson A. D. O'Gara F. (2015). The sound of silence: activating silent biosynthetic gene clusters in marine microorganisms. Mar. Drugs 13 (8), 4754–4783. 10.3390/md13084754 26264003 PMC4557003

[B155] Rigolet A. Arias A. A. Anckaert A. Quinton L. Rigali S. Tellatin D. (2023). The fate of bacterial secondary metabolites in the rhizosphere: Streptomyces degrades and feeds on cyclic lipopeptides produced by competitors. BioRxiv. 10.1101/2023.07.27.550914

[B156] Rokni-Zadeh H. Li W. Sanchez-Rodriguez A. Sinnaeve D. Rozenski J. Martins J. C. (2012). Genetic and functional characterization of cyclic lipopeptide white-line-inducing principle (WLIP) production by rice rhizosphere isolate *Pseudomonas putida* RW10S2. Appl. Environ. Microbiol. 78 (14), 4826–4834. 10.1128/AEM.00335-12 22544260 PMC3416372

[B157] Rokni-Zadeh H. Li W. Yilma E. Sanchez-Rodriguez A. De Mot R. (2013). Distinct lipopeptide production systems for WLIP (white line-inducing principle) in *Pseudomonas fluorescens* and *Pseudomonas putida* . Environ. Microbiol. Rep. 5 (1), 160–169. 10.1111/1758-2229.12015 23757145

[B158] Roongsawang N. Washio K. Morikawa M. (2011). Diversity of nonribosomal peptide synthetases involved in the biosynthesis of lipopeptide biosurfactants. Int. J. Mol. Sci. 12 (1), 141–172. 10.3390/ijms12010141 PMC3039948 21339982

[B159] Rosen P. C. Seyedsayamdost M. R. (2017). Though much is taken, much abides: finding new antibiotics using old ones. Biochemistry 56 (37), 4925–4926. 10.1021/acs.biochem.7b00782 28862834 PMC5617734

[B160] Sanahuja G. Banakar R. Twyman R. M. Capell T. Christou P. (2011). *Bacillus thuringiensis:* a century of research, development and commercial applications. Plant Biotechnol. J. 9 (3), 283–300. 10.1111/j.1467-7652.2011.00595.x 21375687

[B161] Scherlach K. Lackner G. Graupner K. Pidot S. Bretschneider T. Hertweck C. (2013). Biosynthesis and mass spectrometric imaging of tolaasin, the virulence factor of Brown blotch mushroom disease. Chembiochem 14 (18), 2439–2443. 10.1002/cbic.201300553 24222604

[B162] Sekar J. Raju K. Duraisamy P. Vaiyapuri P. R. (2018). Potential of finger millet indigenous rhizobacterium *Pseudomonas* sp. MSSRFD41 in blast disease management - growth promotion and compatibility with the resident rhizomicrobiome. Front. Microbiol. 9, 1029. 10.3389/fmicb.2018.01029 29875748 PMC5974220

[B163] Sekhon Randhawa K. K. Rahman P. K. S. M. (2014). Rhamnolipid biosurfactants-past, present, and future scenario of global market. Front. Microbiol. 5, 454. 10.3389/fmicb.2014.00454 25228898 PMC4151382

[B164] Seyedsayamdost M. R. (2014). High-throughput platform for the discovery of elicitors of silent bacterial gene clusters. Proc. Natl. Acad. Sci. 111 (20), 7266–7271. 10.1073/pnas.1400019111 24808135 PMC4034229

[B165] Shimura S. Ishima M. Nakajima S. Fujii T. Himeno N. Ikeda K. (2013). Total synthesis and anti-hepatitis C virus activity of MA026. J. Am. Chem. Soc. 135 (50), 18949–18956. 10.1021/ja410145x 24251365

[B166] Siani H. S. Barragan-Huerta B. E. Lebron-Paler A. Pemberton J. E. Vazquez R. R. Burns A. M. (2008). Efficient purification of the biosurfactant viscosin from *Pseudomonas libanensis* strain M9-3 and its physicochemical and biological properties. J. Nat. Prod. 71 (6), 1011–10115. 10.1021/np800069u 18471020

[B167] Singh G. M. Fortin P. D. Koglin A. Walsh C. T. (2008). β-Hydroxylation of the aspartyl residue in the phytotoxin syringomycin E: characterization of two candidate hydroxylases AspH and SyrP in *Pseudomonas syringae* . Biochemistry 47 (43), 11310–11320. 10.1021/bi801322z 18826255 PMC2600472

[B168] Singh G. M. Vaillancourt F. H. Yin J. Walsh C. T. (2007). Characterization of SyrC, an aminoacyltransferase shuttling threonyl and chlorothreonyl residues in the syringomycin biosynthetic assembly line. Chem. Biol. 14 (1), 31–40. 10.1016/j.chembiol.2006.11.005 17254950

[B169] Soberón-Chávez G. González-Valdez A. Soto-Aceves M. P. Cocotl-Yañez M. (2021). Rhamnolipids produced by *Pseudomonas*: from molecular genetics to the market. Microb. Biotechnol. 14 (1), 136–146. 10.1111/1751-7915.13700 33151628 PMC7888470

[B170] Sobrero P. M. Muzlera A. Frescura J. Jofré E. Valverde C. (2017). A matter of hierarchy: activation of orfamide production by the post-transcriptional Gac-Rsm cascade of *Pseudomonas protegens* CHA0 through expression upregulation of the two dedicated transcriptional regulators. Environ. Microbiol. Rep. 9 (5), 599–611. 10.1111/1758-2229.12566 28703431

[B171] Solaiman D. K. Ashby R. D. Gunther N. W. t. Zerkowski J. A. (2015). Dirhamnose-lipid production by recombinant nonpathogenic bacterium Pseudomonas chlororaphis. Appl. Microbiol. Biotechnol. 99 (10), 4333–4342. 10.1007/s00253-015-6433-4 25661819

[B172] Soler-Rivas C. Jolivet S. Arpin N. Olivier J. M. Wichers H. J. (1999). Biochemical and physiological aspects of brown blotch disease of *Agaricus bisporus* . FEMS Microbiol. Rev. 23 (5), 591–614. 10.1016/S0168-6445(99)00023-6 10525168

[B173] Song C. Aundy K. van de Mortel J. Raaijmakers J. M. (2014). Discovery of new regulatory genes of lipopeptide biosynthesis in *Pseudomonas fluorescens* . FEMS Microbiol. Lett. 356 (2), 166–175. 10.1111/1574-6968.12404 25202778

[B174] Song C. Sundqvist G. Malm E. de Bruijn I. Kumar A. van de Mortel J. (2015a). Lipopeptide biosynthesis in P*seudomonas fluorescens* is regulated by the protease complex ClpAP. BMC Microbiol. 15 (1), 29–367. 10.1186/s12866-015-0367-y 25885431 PMC4332742

[B175] Song C. van der Voort M. van de Mortel J. Hassan K. A. Elbourne L. D. H. Paulsen I. T. (2015b). The Rsm regulon of plant growth-promoting *Pseudomonas fluorescens* SS101: role of small RNAs in regulation of lipopeptide biosynthesis. Microb. Biotechnol. 8 (2), 296–310. 10.1111/1751-7915.12190 25488342 PMC4353343

[B176] Sonnleitner E. Haas D. (2011). Small RNAs as regulators of primary and secondary metabolism in *Pseudomonas* species. Appl. Microbiol. Biotechnol. 91 (1), 63–79. 10.1007/s00253-011-3332-1 21607656

[B177] Sorensen K. N. Kim K. H. Takemoto J. Y. (1996). *In vitro* antifungal and fungicidal activities and erythrocyte toxicities of cyclic lipodepsinonapeptides produced by *Pseudomonas syringae* pv syringae. Antimicrob. Agents Chemother. 40 (12), 2710–2713. 10.1128/Aac.40.12.2710 9124827 PMC163608

[B178] Spraker J. E. Sanchez L. M. Lowe T. M. Dorrestein P. C. Keller N. P. (2016). Ralstonia solanacearum lipopeptide induces chlamydospore development in fungi and facilitates bacterial entry into fungal tissues. ISME J. 10 (9), 2317–2330. 10.1038/ismej.2016.32 26943626 PMC4989320

[B179] Steigenberger J. Mergen C. De Roo V. Geudens N. Martins J. C. Heerklotz H. (2023). The effect of membrane thickness on the membrane permeabilizing activity of the cyclic lipopeptide tolaasin II. Front. Mol. Biosci. 9, 1064742. 10.3389/fmolb.2022.1064742 PMC9817028 36619163

[B180] Steigenberger J. Verleysen Y. Geudens N. Martins J. C. Heerklotz H. (2021). The optimal lipid chain length of a membrane-permeabilizing lipopeptide results from the balance of membrane partitioning and local damage. Front. Microbiol. 12, 669709–669714. 10.3389/fmicb.2021.669709 34594308 PMC8476953

[B181] Sun W. Zhu B. Yang F. Dai M. Sehar S. Peng C. (2021). Optimization of biosurfactant production from Pseudomonas sp. CQ2 and its application for remediation of heavy metal contaminated soil. Chemosphere 265, 129090. 10.1016/j.chemosphere.2020.129090 33293052

[B182] Tavares L. F. Silva P. M. Junqueira M. Mariano D. C. Nogueira F. C. Domont G. B. (2013). Characterization of rhamnolipids produced by wild-type and engineered *Burkholderia kururiensis* . Appl. Microbiol. Biotechnol. 97 (5), 1909–1921. 10.1007/s00253-012-4454-9 23053103

[B183] Temme K. Zhao D. Voigt C. A. (2012). Refactoring the nitrogen fixation gene cluster from Klebsiella oxytoca. Proc. Natl. Acad. Sci. U. S. A. 109 (18), 7085–7090. 10.1073/pnas.1120788109 22509035 PMC3345007

[B184] Tran H. Ficke A. Asiimwe T. Höfte M. Raaijmakers J. M. (2007). Role of the cyclic lipopeptide massetolide a in biological control of *Phytophthora infestans* and in colonization of tomato plants by *Pseudomonas fluorescens* . New Phytol. 175 (4), 731–742. 10.1111/j.1469-8137.2007.02138.x 17688588

[B185] Vallet-Gely I. Novikov A. Augusto L. Liehl P. Bolbach G. Péchy-Tarr M. (2010). Association of hemolytic activity of *Pseudomonas entomophila,* a versatile soil bacterium, with cyclic lipopeptide production. Appl. Environ. Microbiol. 76 (3), 910–921. 10.1128/AEM.02112-09 20023108 PMC2812987

[B186] Van Der Voort M. Meijer H. J. G. Schmidt Y. Watrous J. Dekkers E. Mendes R. (2015). Genome mining and metabolic profiling of the rhizosphere bacterium *Pseudomonas* sp. SH-C52 for antimicrobial compounds. Front. Microbiol. 6, 693. 10.3389/fmicb.2015.00693 26217324 PMC4493835

[B187] Vaughn V. L. Gross D. C. (2016). Characterization of salA, syrF, and syrG genes and attendant regulatory networks involved in plant pathogenesis by *Pseudomonas syringae* pv. syringae B728a. PLoS One 11 (3), e0150234. 10.1371/journal.pone.0150234 26954255 PMC4783005

[B188] Venkatesh N. Greco C. Drott M. T. Koss M. J. Ludwikoski I. Keller N. M. (2022). Bacterial hitchhikers derive benefits from fungal housing. Curr. Biol. 32 (7), 1523–1533.e6. 10.1016/j.cub.2022.02.017 35235767 PMC9009100

[B189] Venturi V. (2006). Regulation of *quorum* sensing in *Pseudomonas* . FEMS Microbiol. Rev. 30 (2), 274–291. 10.1111/j.1574-6976.2005.00012.x 16472307

[B190] Wang N. Lu S.-E. Records A. R. Gross D. C. (2006a). Characterization of the transcriptional Activators SalA and SyrF, which are required for syringomycin and syringopeptin production by *Pseudomonas syringae* pv. *syringae* . J. Bacteriol. 188 (9), 3290–3298. 10.1128/JB.188.9.3290-3298.2006 16621822 PMC1447436

[B191] Wang N. Lu S. E. Wang J. L. Chen Z. J. Gross D. C. (2006b). The expression of genes encoding lipodepsipeptide phytotoxins by *Pseudomonas syringae* pv. *syringae* is coordinated in response to plant signal molecules. Mol. Plant-Microbe Interact. 19 (3), 257–269. 10.1094/Mpmi-19-0257 16570656

[B192] Wang S. Cui J. Bilal M. Hu H. Wang W. Zhang X. (2020). *Pseudomonas* spp. as cell factories (MCFs) for value-added products: from rational design to industrial applications. Crit. Rev. Biotechnol. 40 (8), 1232–1249. 10.1080/07388551.2020.1809990 32907412

[B193] Wang W. Zheng G. Lu Y. (2021). Recent advances in strategies for the cloning of natural product biosynthetic gene clusters. Front. Bioeng. Biotechnol. 9, 692797. 10.3389/fbioe.2021.692797 34327194 PMC8314000

[B194] Washio K. Lim S. P. Roongsawang N. Morikawa M. (2010). Identification and characterization of the genes responsible for the production of the cyclic lipopeptide arthrofactin by *Pseudomonas* sp. MIS38. Biosci. Biotechnol. Biochem. 74 (5), 992–999. 10.1271/bbb.90860 20460722

[B195] Whiteley M. Greenberg E. P. (2001). Promoter specificity elements in *Pseudomonas aeruginosa quorum*-sensing-controlled genes. J. Bacteriol. 183 (19), 5529–5534. 10.1128/Jb.183.19.5529-5534.2001 11544214 PMC95443

[B196] Wirth N. T. Kozaeva E. Nikel P. I. (2020). Accelerated genome engineering of Pseudomonas putida by I-SceI-mediated recombination and CRISPR-Cas9 counterselection. Microb. Biotechnol. 13 (1), 233–249. 10.1111/1751-7915.13396 30861315 PMC6922521

[B197] Woo S. Fogliano V. Scala F. Lorito M. (2002). Synergism between fungal enzymes and bacterial antibiotics may enhance biocontrol. Ant. Van Leeuwenhoek 81 (1-4), 353–356. 10.1023/a:1020540818163 12448733

[B198] Xia B. Luo M. Pang L. Liu X. Yi Y. (2021). Lipopeptides against COVID-19 RNA-dependent RNA polymerase using molecular docking. Biomed. J. 44 (6 Suppl. 1), S15–s24. 10.1016/j.bj.2021.11.010 34871815 PMC8641408

[B199] Yeh M. S. Wei Y. H. Chang J. S. (2005). Enhanced production of surfactin from *Bacillus subtilis* by addition of solid carriers. Biotechnol. Progr 21 (4), 1329–1334. 10.1021/bp050040c 16080719

[B200] Zachow C. Jahanshah G. De Bruijn I. Song C. Ianni F. Pataj Z. (2015). The novel lipopeptide poaeamide of the endophyte *Pseudomonas poae* RE-1-1-14 is involved in pathogen suppression and root colonization. Mol. Plant-Microbe Interact. 28 (7), 800–810. 10.1094/MPMI-12-14-0406-R 25761208

[B201] Zhang S. Mukherji R. Chowdhury S. Reimer L. Stallforth P. (2021). Lipopeptide-mediated bacterial interaction enables cooperative predator defense. Proc. Natl. Acad. Sci. 118 (6), e2013759118. 10.1073/pnas.2013759118 33526668 PMC8017672

[B202] Zhao F. Wu Y. Wang Q. Zheng M. Cui Q. (2021). Glycerol or crude glycerol as substrates make *Pseudomonas aeruginosa* achieve anaerobic production of rhamnolipids. Microb. Cell Factories 20 (1), 185. 10.1186/s12934-021-01676-2 PMC8461908 34556134

[B203] Zhao H. Liu Y. P. Zhang L. Q. (2019). *In silico* and genetic analyses of cyclic lipopeptide synthetic gene clusters in *Pseudomonas* sp. 11K1. Front. Microbiol. 10, 544–611. 10.3389/fmicb.2019.00544 30941113 PMC6433849

[B204] Zhou L. de Jong A. Yi Y. H. Kuipers O. P. (2021). Identification, isolation, and characterization of medipeptins, antimicrobial peptides from *Pseudomonas mediterranea* EDOX. Front. Microbiol. 12, 732771. 10.3389/fmicb.2021.732771 34594316 PMC8477016

[B205] Zhu H. Sandiford S. K. van Wezel G. P. (2014). Triggers and cues that activate antibiotic production by actinomycetes. J. Industrial Microbiol. Biotechnol. 41 (2), 371–386. 10.1007/s10295-013-1309-z 23907251

[B206] Zong G. Fu J. Zhang P. Zhang W. Xu Y. Cao G. (2022). Use of elicitors to enhance or activate the antibiotic production in *Streptomyces* . Crit. Rev. Biotechnol. 42 (8), 1260–1283. 10.1080/07388551.2021.1987856 34706600

